# Results of the home mechanical ventilation national program among adults in Chile between 2008 and 2017

**DOI:** 10.1186/s12890-021-01764-4

**Published:** 2021-12-02

**Authors:** César Maquilón, Mónica Antolini, Nicolás Valdés, Marianela Andrade, Krishnna Canales, Claudio Rabec, Cristian Olave, Miguel Aguayo, Patricia Rivas, Carmen Andrade, Ángela Venegas, Sandra Zapata, María Elena Torres, Osvaldo Cabrera, Jorge Villalobos

**Affiliations:** 1 Department of Respiratory Diseases, Clínica Dávila, Recoleta 464, Building H, 6th floor, Santiago, Chile; 2 Ministry of Health, AVNIA-AVIA Programs, Santiago, Chile; 3 School of Nursing and Obstetrics, Universidad de los Andes, Santiago, Chile; 4 Service de Pneumologie Et Soins Intensifs Respiratoires, CHU Dijon Bourgogne, Dijon, France; 5 Instituto Nacional del Tórax, Santiago, Chile

**Keywords:** Home mechanical ventilation, Obesity hypoventilation syndrome, Chronic respiratory failure, Domiciliary ventilation, Long term ventilation

## Abstract

**Background:**

Home mechanical ventilation (HMV) is a viable and effective strategy for patients with chronic respiratory failure (CRF). The Chilean Ministry of Health started a program for adults in 2008.

**Methods:**

This study examined the following data from a prospective cohort of patients with CRF admitted to the national HMV program: characteristics, mode of admission, quality of life, time in the program and survival.

**Results:**

A total of 1105 patients were included. The median age was 59 years (44–58). Women accounted for 58.1% of the sample. The average body mass index (BMI) was 34.9 (26–46) kg/m^2^. A total of 76.2% of patients started HMV in the stable chronic mode, while 23.8% initiated HMV in the acute mode. A total of 99 patients were transferred from the children's program. There were 1047 patients on non-invasive ventilation and 58 patients on invasive ventilation. The median baseline PaCO_2_ level was 58.2 (52–65) mmHg. The device usage time was 7.3 h/d (5.8–8.8), and the time in HMV was 21.6 (12.2–49.5) months. The diagnoses were COPD (35%), obesity hypoventilation syndrome (OHS; 23.9%), neuromuscular disease (NMD; 16.3%), non-cystic fibrosis bronchiectasis or tuberculosis (non-CF BC or TBC; 8.3%), scoliosis (5.9%) and amyotrophic lateral sclerosis (ALS; 5.24%). The baseline score on the Severe Respiratory Insufficiency questionnaire (SRI) was 47 (± 17.9) points and significantly improved over time. The lowest 1- and 3-year survival rates were observed in the ALS group, and the lowest 9-year survival rate was observed in the non-CF BC or TB and COPD groups. The best survival rates at 9 years were OHS, scoliosis and NMD. In 2017, there were 701 patients in the children's program and 722 in the adult´s program, with a prevalence of 10.4 per 100,000 inhabitants.

**Conclusion:**

The most common diagnoses were COPD and OHS. The best survival was observed in patients with OHS, scoliosis and NMD. The SRI score improved significantly in the follow-up of patients with HMV. The prevalence of HMV was 10.4 per 100,000 inhabitants.

*Trial registration* This study was approved by and registered at the ethics committee of North Metropolitan Health Service of Santiago, Chile (N° 018/2021).

**Supplementary Information:**

The online version contains supplementary material available at 10.1186/s12890-021-01764-4.

## Background

Home mechanical ventilation (HMV) is a viable and effective treatment strategy for patients with chronic respiratory failure (CRF). HMV has been used since the 1980s, and in recent decades, its use has increased for a wide range of diseases, including neuromuscular diseases [1], restrictive thoracic diseases, obesity hypoventilation syndrome (OHS) [2, 3] and advanced chronic obstructive pulmonary disease (COPD) [4]. HMV seeks to correct hypoventilation, relieve symptoms, decrease hospitalizations, and improve quality of life and survival [5, 6]. The prevalence of HMV reported in Europe 20 years ago was 6.6 per 100,000 inhabitants [7], but currently, it has increased due to obesity and COPD. A study carried out by ANTADIR in France describes recent changes in the causes of CRF and mentions that OHS is an important indication for the use of HMV [8]. A Canadian study reported that COPD and OHS are the most frequent diagnoses for admission into an HMV program among 4670 patients [9]. In a region of Switzerland, Cantero et al. described how over two decades, the incidence of COPD- and OHS-induced HMV has increased to 37.9 per 100,000 inhabitants [10]. In Chile, there are no reports of the prevalence of HMV, but the prevalence of diseases that increase the risk of CRF, such as obesity (34.4%) and smoking (33.3%), is known [11]. The Chilean health system is mixed, with public health insurance covering 78% of the country's population, private health insurance covering 14.4% and armed forces covering 3% [12]. In 2006, the Ministry of Health of Chile (Ministerio de Salud de Chile—MINSAL) initiated a program of invasive and non-invasive ventilation in children under 20 years of age covered by public health insurance [13]. In 2008, the MINSAL initiated a non-invasive home ventilation program for adults older than 20 years with CRF for multiple causes (AVNIA program, for its acronym in Spanish), and in 2014, invasive mechanical ventilation through tracheostomy was included in the program [14].

The MINSAL proposed the following goals: (a) provide CRF patients with the technology and trained personnel for periodic home supervision, focusing on family and self-care; (b) reduce the mean number of days of hospitalization per year and vacate intensive care unit beds to allow the admission of patients with acute diseases; and (c) improve quality of life by focusing on social reintegration.

## Methods

### Study design and patients

The following data were collected from a prospective cohort of adult patients with CRF consecutively admitted to the national HMV program: demographic, clinical and functional characteristics according to diagnostic groups; modes of admission; quality of life; length of stay in the program; causes of discharge; and survival.

All patients of both sexes and over 20 years of age with CRF admitted between May 1, 2008, and December 31, 2017, to the State HMV program, hospitals, and primary care clinics in 10 regions of Chile were included in the study. The two modes of admission were acute hospitalized patients (immediately after discharge from hospitalization for exacerbation) or stable chronic patients (electively from outpatient monitoring). In both cases, the patient was evaluated by the program doctor [14] who approved or refused admission according to the established inclusion criteria in the program (Additional file 1, technical standard for HMV programs. Ministry of Health, versions 2008, 2012 and 2013, Chile.doc).

### Inclusion criteria and diagnostic groups

For NIV indications in patients at steady state, the ACCP Consensus Conference criteria were applied [15]. For obesity hypoventilation (OHS), thoracic cage disorders (TCDs) and neuromuscular diseases (NMDs), long-term NIV was indicated in patients with daytime hypercapnia > 45 mmHg associated with symptoms of hypoventilation. COPD patients were considered for HMV initiation when they also had at least 1 hospitalization in the last 12 months, PaCO_2_ levels > 55 mmHg in stable condition with > 30 days after the last exacerbation or with PaCO_2_ levels > 50 mmHg associated with deep and numerous nocturnal desaturations. These criteria were the same as those applied to patients grouped as presenting with sequelae of non-cystic fibrosis bronchiectasis or tuberculosis (non-CF BC or TB). (Page 10, Additional file 1).

The pathologies were ultimately grouped as follows: COPD, OHS, non-CF BC or TB, ALS, non-ALS neuromuscular diseases (NMDs), scoliosis and others.

### Exclusion criteria

The exclusion criteria were absence of a family support network, a home lacking the minimum required conditions (lack of electricity or plumbing), active smoking and drug addiction (Page 11, Additional file 1).

### Data collected

The following were recorded prospectively: sex; age; BMI; spirometry; baseline daytime arterial gases (room air or oxygen supply in patients needing supplementary oxygen); rural or urban residence; region of the country; section of the public health insurance (state health insurance classifies its insured according to their income level, the lower the income, the more coverage the state insurance provides); family APGAR (assessed adult satisfaction with social support from the family, a score of 7–10 suggests a highly functional family) [16, 17]; baseline, 6, 12 and 36 months score on the Severe Respiratory Insufficiency (SRI) questionnaire; mode of admission (acute hospitalized patient, chronic stable patient or transferred from the children’s program); use of home oxygen; interface (non-invasive or tracheostomy); spontaneous (S), spontaneous/timed (S/T), hybrid [average volume assured pressure support (AVAPS) and intelligent volume-assured pressure support (iVAPS)] or other ventilation modes (pressure control, volume control, synchronous intermittent mandatory ventilation); and ventilator parameters.

From the time of admission to the program, the patient was followed up by the assigned program’s physician at one month, at three months, and then every six months until year 4, after which the follow-up was performed annually if the patients remained stable. Patients were regularly visited at their home 1–3 times a week by a physiotherapist and once a month by a nurse. These professionals were responsible for the continuing education of the patient and caregiver, the application of surveys, facial skin care, following test protocols and data collection.

The following data were collected at each visit: interface type and ventilator type, state of the equipment, mask-related complications, pulmonary function tests [FEV1, VC, maximal inspiratory pressure (MIP), maximal expiratory pressure (MEP), Sniff nasal inspiratory pressure], baseline spirometry including basal and postbronchodilator tests and predictive values according to Knudson [18]. The data were obtained from devices built in the monitoring system (days of usage, average hours per day, average pressures, leaks). ABG and/or transcutaneous CO_2_ capnography were performed with the usual flow of O_2_ used. An overnight pulse oximetry was also performed, and the following information was collected: mean and lowest saturation, time spent with SpO_2_ 90% (CT90), and oxygen desaturation index (Page 24 to 27, Additional file 1). All collected data were updated for each visit in the database (respiratorio.minsal.cl.).

The causes for discharge from the program were grouped as follows: poor adherence (defined as using the ventilator for less than 4 h a day for 3 months); other modes of non-compliance (non-attendance at medical check-ups, repeated absences at home when trying to visit him or her); voluntary withdrawal; transfer to another program; and improvement with exit from the program.

The survival analysis was conducted until August 1, 2018. This study was approved by the ethics committee of North Metropolitan Health Service of Santiago (Servicio de Salud Metropolitano Norte de Santiago), informed consent was obtained, and the study was conducted in accordance with the Declaration of Helsinki.

### Health-related quality of life (HRQL)

Quality of life was evaluated with the Spanish version of the SRI questionnaire [19, 20]. Preliminary data from a Chilean version of this questionnaire showed good reliability compared to the original data [21].

### Statistical analysis

The quantitative variables were expressed as the mean and standard deviation (SD) for those with a normal distribution and as the median and interquartile range (IQR1, IQR3) for those with a nonnormal distribution. Categorical variables were expressed as absolute and relative frequencies. Differences were estimated with ANOVA for numerical variables and with the chi^2^ test for categorical variables. Kaplan–Meier curves were used for the survival analysis with a closing date of August 1, 2018. The data were entered and analysed using the program STATA 14.2 IC (StataCorp LLC, USA).

## Results

### Patient characteristics, diagnostic groups and time spent in the program

In the described period, 2127 patients were recruited to the program through the website of the MINSAL HMV programs. A total of 1022 (48%) patients did not enter the program. Among those who did not enter the program, 541 (53%) were men, the average age was 59.5 (± 17.9) years, and 53.3% of the rejected patients lived in Santiago (metropolitan region). The main reasons for not entering the program were as follows: patient did not meet arterial blood gas criteria (49.4%), refused to be included, did not attend the medical appointment or no contact is achieved (17%), died before contact (6.3%), purchased equipment and services on their own (4%), active smoking or drug addiction (4%), and other causes (19.3%) (see Annex 2: Interconsultation for AVNIA PRE-ADMISSION evaluation, pages 32 to 34. Additional file 1).

As of December 31, 2017, 1105 patients were consecutively admitted. The median age (IQR) was 59 (44–58) years; 58.1% were women; 762 (68.9%) lived in Santiago (metropolitan region); and 343 (31.1%) lived in other regions of the country. The median BMI was 34.9 (26–46) kg/m^2^ (Table 1).

**Table 1:** Baseline demographic and clinical characteristics of HMV patients in Chile (n = 1105)

*Characteristics*		
Female	n (%)	572 (51.8)
Male	n (%)	533 (48.2)
Median age	Median [IQR]	59 (44–68)
BMI (n = 526)	Median [IQR]	34.9 (26–46)
*Residence*		
Urban (n) (%)	n (%)	1088 (98.5)
Rural (n) (%)	n (%)	17 (1.5)
*State health insurance (according to monthly income in 2020)*		
Grupo A and B (450 USD)	n (%)	942 (85.4)
Grupo C (> 450 y ≤ 657 USD)	n (%)	82 (7.4)
Group D (> 657 USD)	n (%)	79 (7.2)
Family APGAR (n = 435)	Median [IQR]	10 (8–10)
Baseline overall SRI Score	Median [IQR]	47 (35–62.1)
*Comorbidities*		
Arterial hypertension	n (%)	710 (64.5)
Diabetes	n (%)	354 (32.2)
*Initiation of ventilation*		
Acute	n (%)	263 (23.8)
Chronic	n (%)	842 (76.2)
Transferred from the children's program	n (%)	99 (8.9)
Patients with ≤ 90 days	n (%)	74 (6.7)
Patients with > 90 days	n (%)	1031 (93.3)
*Diagnosis causing hypoventilation (Groups)*		
(1) COPD	n (%)	388 (35)
(2) OHS		264 (23.9)
(3) NMD		180 (16.3)
(4) non-CF BC or TBC		92 (8.3)
(5) SCOLIOSIS		65 (5.9)
(6) ALS		58 (5.24)
(7) OTHER DIAGNOSES		58 (5.24)
*Lung function (Spirometry) (n = 623)*		
FVC (L)	Median [IQR]	1.53 (1.06–2.07)
FEV_1_ (L)	Median [IQR]	0.88 (0.6–1.42)
FEV_1_/FVC (%)	Median [IQR]	71 (51–84)
*Arterial blood gas*		
p_a_O_2_ (mmHg)	Median [IQR]	62 (54–72)
p_a_CO_2_ (mmHg)	Median [IQR]	58.2 (52–65)
HCO_3_^−^ (mmol/L)	Median [IQR]	32 (28–35)

*HMV* home mechanical ventilation, *BMI* body mass index, *URBAN* area with > 10,000 people, *APGAR* screening for family dysfunction, 7–10 suggests a highly functional family, *SRI* severe respiratory insufficiency score, *COPD* chronic obstructive pulmonary disease, *OHS* obesity hypoventilation syndrome, *NMD* neuromuscular disease, *non-CF BC or TB* non-cystic fibrosis bronchiectasis or tuberculosis, *ALS* amyotrophic lateral sclerosis, *OTHER DIAGNOSES* cystic fibrosis, pulmonary fibrosis, phrenic paralysis, posttransplant bronchiolitis, *FVC* forced vital capacity, *FEV*_*1*_ forced expiratory volume in 1 s

A total of 98.5% of the patients lived in urban areas. A total of 76.2% (842 patients) started HMV in the chronic stable mode and 23.8% (263 patients) in the acute mode. A total of 99 patients were transferred from the children's program to the adult program.

The underlying diagnoses leading to NIV initiation were as follows: COPD, 388 patients (35%); OHS, 264 patients (23.9%); NMD, 180 patients (16.3%); non-CF BC or TB, 92 patients (8.3%); scoliosis, 65 patients (5.9%); ALS, 58 patients (5.24%); and other diagnoses, 58 patients (5.24%) (Table 1). The median baseline paCO_2_ level of the overall sample was 58.2 (52–65) mmHg (Table 1).

**Fig. 1:**
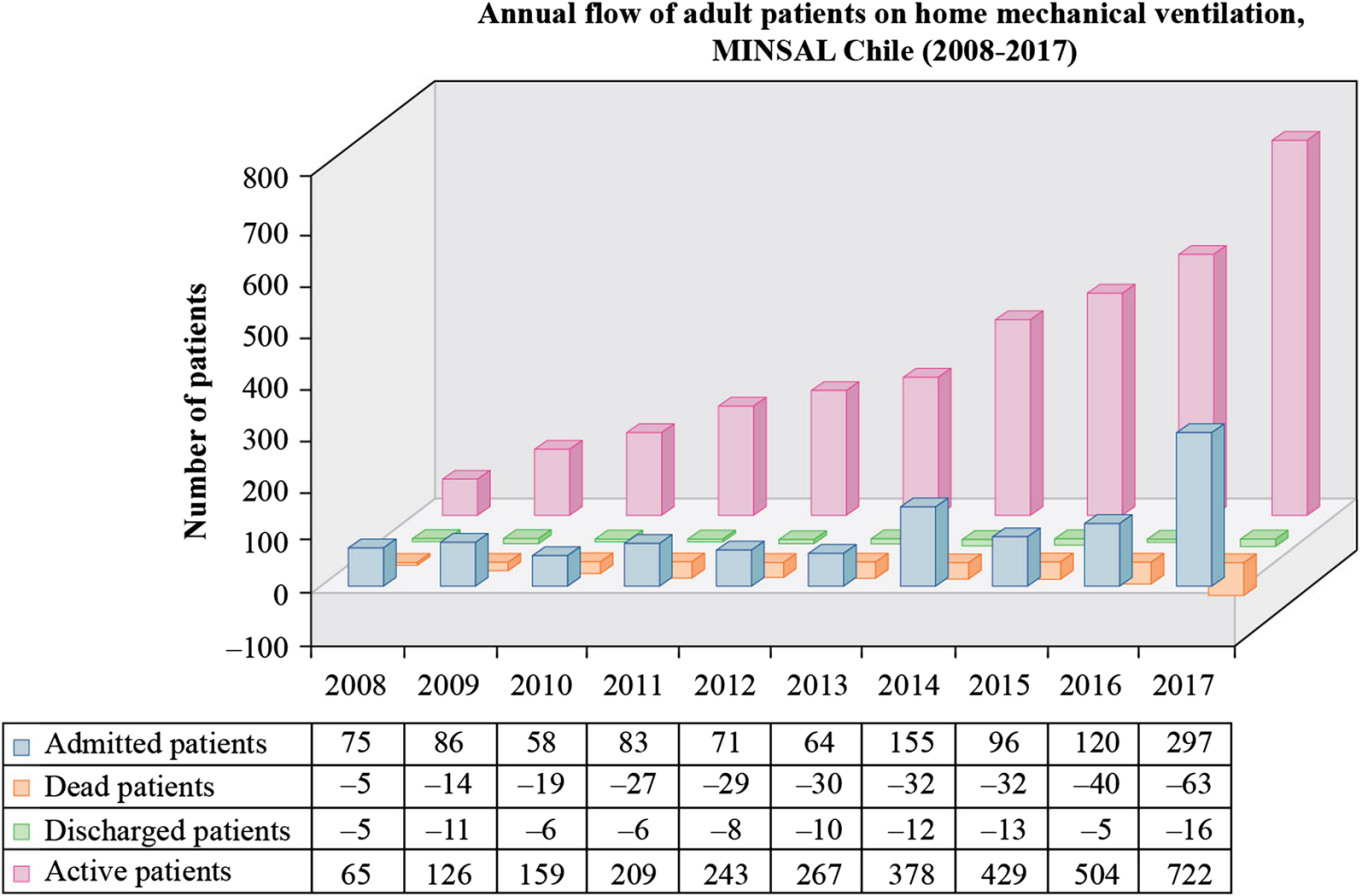
Annual flow of patients in home mechanical ventilation programs from 2008 to 2017 (n = 1105)

**Fig. 2:**
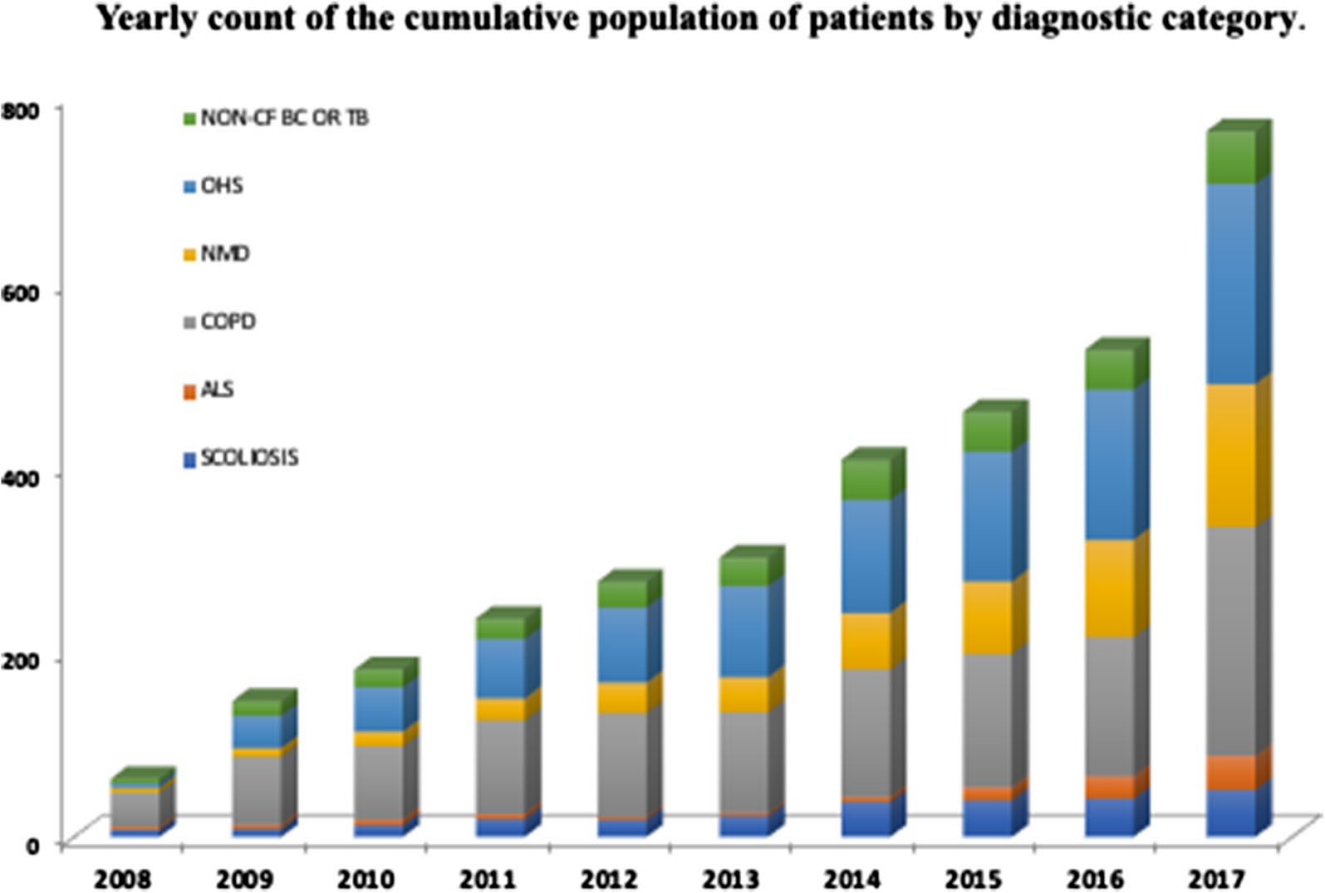
Yearly count of the cumulative population of patients treated with HMV (2008–2017)

The program expanded to other regions of the country, and the number of active patients per year increased, as well as the percentage of NMD patients (Figs. 1, 2). The mean length in the program was 21.6 (12.2–49.5) months, with the longest duration being observed in the scoliosis group and the shortest duration being observed in the ALS group, at 46.1 (± 33.3) months and 14.8 (± 10.4) months, respectively.

### Home mechanical ventilation characteristics

A total of 1047 (94.8%) patients were ventilated noninvasively, and 58 (5.24%) were ventilated invasively (Table 2). The most common ventilatory mode was S/T (86.8%). The median baseline IPAP level was 16 (14–18) cmH_2_O. Patients were ventilated for 7.3 (5.8–8.8) hours per day (Table 2).

**Table 2:** Baseline ventilatory characteristics of HMV patients in Chile (n = 1105)

O_2_ supplementation need (LTOT)	n (%)	688 (62.4)
*Interface*		
Non-invasive	n (%)	1047 (94.8)
Invasive (tracheostomy)	N (%)	58 (5.2)
Time in home mechanical ventilation (months)	Median [IQR]	21.6 (12.2–49.5)
*Ventilatory modes (n* = *706 patients)*		
Spontaneous/timed (S/T)	n (%)	613 (86.8)
Spontaneous (S)	n (%)	34 (4.8)
Hybrid (AVAPS or iVAPS)	n (%)	46 (6.5)
Other modes	n (%)	13 (1.8)
Compliance (hours/day)	Median [IQR]	7.3 (5.8–8.8)
*Ventilator settings (n* = *706 patients)*		
IPAP (cmH_2_O)	Median [IQR]	16 (14–18)
EPAP (cmH_2_O)	Median [IQR]	6 (6–8)
Maximum IPAP (cmH_2_O) (only hybrid mode)	Median [IQR]	18 (16–22)
Backup respiratory rate	Median [IQR]	15 (14–16)

*HMV* home mechanical ventilation, *LTOT* long time oxygen therapy, *AVAPS* average volume assured pressure support, *iVAPS* intelligent volume-assured pressure support, *OTHER MODES* controlled assist mode, synchronous intermittent mandatory ventilation, pressure control and volume control, *IPAP* inspiratory positive airway pressure, *EPAP* expiratory positive airway pressure

The ALS group had the highest percentage of patients ventilated through tracheostomy (29.3%), and in this same group, 44.7% of the patients used HMV for more than 16 h a day (Table 3). As expected, the mean age at inclusion was lowest in NMD patients, and the median BMI was higher among OHS patients. Scoliosis patients had the lowest FVC (Table 3).

**Table 3:** Baseline variables of active, dead, and discharged patients as of August 1, 2018, (n = 1105)

		COPD	OHS	NMD	Non-CF BC or TB	SCOLIOSIS	ALS	Other Diagnoses
n		388	264	180	92	65	58	58
AGE (years)	mean (SD)	65.6 (10)	56.8 (14.4)	31.5 (15.1)	52.4 (19.1)	53.3 (19)	55.5 (12.1)	53.7 (20.5)
FEMALE	n (%)	195 (50.3)	157 (59.5)	73 (40.6)	56 (60.9)	40 (61.5)	19 (32.8)	32(55.2)
BMI	mean (SD)	33.9 (10.1)	47.3 (9.6)	22.5 (6.8)	30.3 (8.8)	27.1 (8.4)	23 (4.4)	29.6(7.9)
*Patients progression*								
ACTIVE	n (%)	203 (52.3)	196 (74.2)	136 (75.6)	43 (46.7)	49 (75.4)	22 (37.9)	26 (44.8)
DEAD		144 (37.1)	39 (14.8)	37 (20.6)	38 (41.3)	13 (20)	31 (53.5)	27 (46.6)
DISCHARGED		41 (10.6)	29 (11)	7 (3.9)	11 (12)	3 (4.6)	5 (8.6)	5 (8.6)
*Lung function*								
FVC (%)	mean (SD)	59.2 (19.4)	69.1 (20.7)	36.7 (25.2)	49.7 (14.5)	36.1 (15.0)	45.5 (12.1)	46.2 (19.6)
FEV_1_ (%)		37.2 (18.5)	67.7 (21.3)	38.2 (25.3)	32.9 (12.4)	34.0 (13.0)	48.0 (14.3)	37.5 (20.4)
FEV_1_/FVC (%)		49,7 (16.3)	79 (10.1)	89.4 (13)	55.2 (14.2)	81.3(12.2)	83 (10.3)	67.1 (20.5)
*Arterial blood gas*								
p_a_O_2_ (mmHg)	mean (SD)	59.9 (14.1)	64 (15)	78.1 (21.2)	61 (14.4)	66.3 (17.7)	80.2 (17.8)	73.7 (25.8)
p_a_CO_2_ (mmHg)		61 (10)	59.2 (10.9)	53.9 (14.6)	61.5 (10.4)	61.9 (12.8)	53.5 (16.7)	57.1 (11.4)
HCO_3_^−^ (mmol/L)		32.7 (5.6)	31.6 (6.5)	29.2 (6.2)	33.5 (5)	34.1 (9.9)	28.5 (4.8)	32.9 (4.3)
*Type of ventilation*								
NIV	n (%)	388 (100)	264 (100)	142 (78.9)	92 (100)	64 (98.5)	41 (70.7)	55 (94.8)
TIV		0	0	38 (21.1)	0	1 (1.54)	17(29.3)	3 (5.17)
Time in HMV (months)	mean (SD)	32 (29.7)	42.3 (32.4)	32.5 (26.4)	34.7 (30.9)	46.1(33.3)	14.8 (10.4)	22.9 (22)
*Compliance (h/day)*								
0–8	(%)	70.8	85.5	44.6	61	73.3	18.42	67.9
> 8 hasta 16		27.9	14.5	38.8	39	24.4	36.84	25
> 16		1.3	0	16.5	0	2.2	44.74	7.14
*Ventilator settings*								
IPAP	mean (SD)	16.6 (3.3)	17.7 (3)	16.5 (4.7)	17.2 (3.6)	15.1 (3.3)	15.5 (4.2)	17.1 (3.9)
EPAP		7.5 (1.3)	7.9 (1.4)	6.5 (1.3)	7.6 (1.4)	6.6(1,4)	6.6 (1.3)	7.4 (1.3)
Backup frequency rate		15.5 (2.5)	15.2 (2.1)	15 (3.1)	14.7 (2.3)	15.3(2.5)	16.4 (3.9)	15.4 (2.9)

*COPD* chronic obstructive pulmonary disease, *OHS* obesity hypoventilation syndrome, *NMD* neuromuscular disease, *non-CF BC or TB* non-cystic fibrosis bronchiectasis or tuberculosis, *ALS* amyotrophic lateral sclerosis, *OTHER DIAGNOSES* cystic fibrosis, pulmonary fibrosis, phrenic paralysis, posttransplant bronchiolitis, *BMI* body mass index, *FVC* forced vital capacity, *FEV*_*1*_ forced expiratory volume in 1 s, *NIV* non-invasive ventilation, *TIV* tracheostomy invasive ventilation, *HMV* home mechanical ventilation, *IPAP* inspiratory positive airway pressure, *EPAP* expiratory positive airway pressure

Regarding NIV initiation in patients ventilated in the steady state, at the beginning of the program (2008–2011), NIV was predominantly introduced in the hospital (mean stay of 3 days), but a gradual switch to NIV initiation at home was observed starting in 2012. Therefore, in recent years, NIV has been initiated at home by a physiotherapist and/or a nurse under the supervision of the assigned program's physician. The average inspiratory positive airway pressure (IPAP) and expiratory positive airway pressure (EPAP) levels programmed at the beginning of ventilatory support were 16.6 (± 3.4) and 7.3 (± 1.4) cmH2O, which were selected to prioritize patient adherence. At 3 months, the average IPAP and EPAP levels were increased to 17.7 (± 3.4) and 7.6 (± 1.5) cmH2O, respectively (*p* < 0.001).

**Fig. 3:**
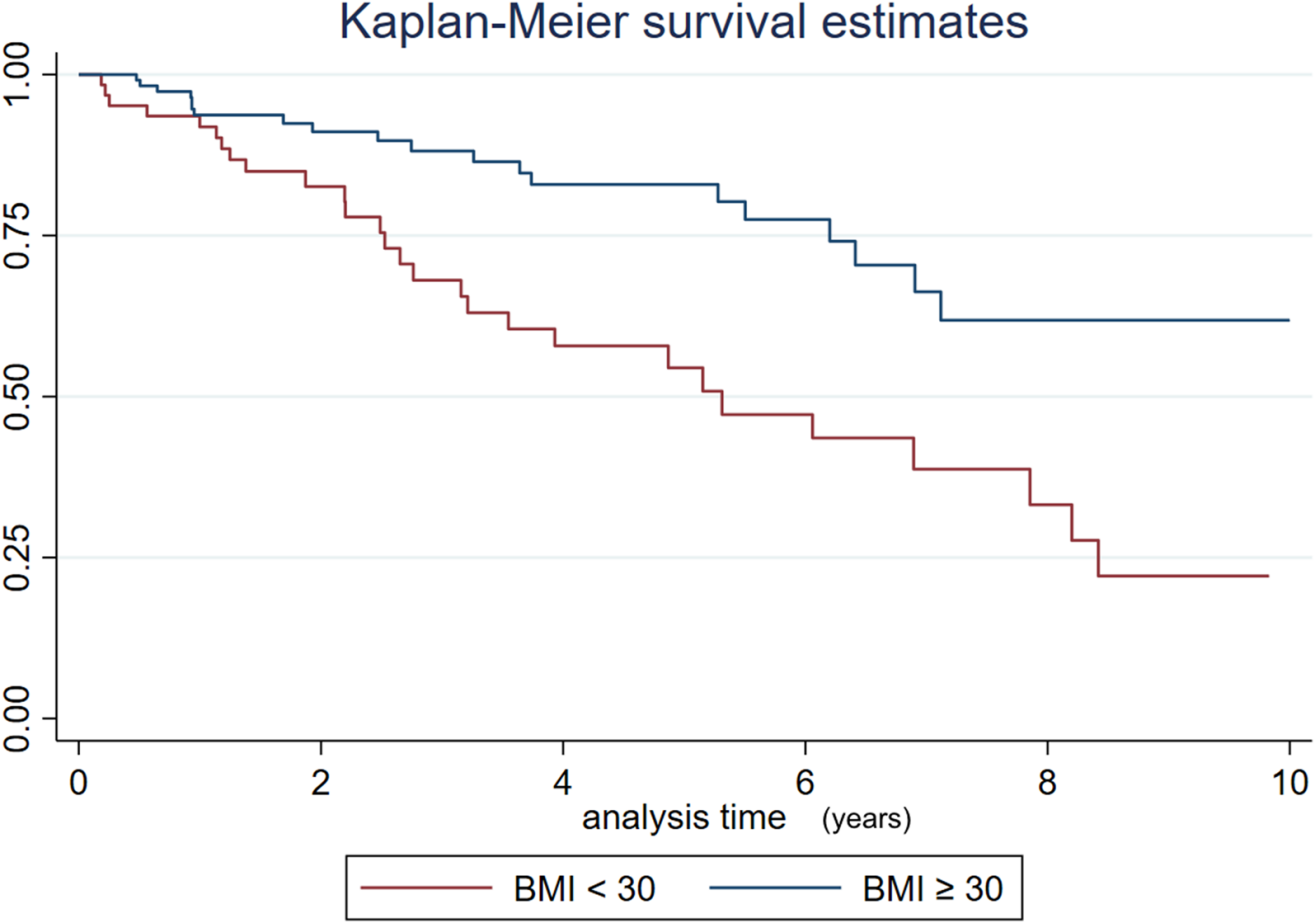
Survival time in years for COPD patients categorized by BMI

In the group of patients with COPD, we only analysed the patients classified as “ACTIVE" or "DECEASED". Of them, we examined patients who had complete functional study and anthropometric data at the time of admission to the program (n = 176). We grouped them according to BMI < 30 or BMI ≥ 30 and compared multiple variables between both groups. There was a significant difference in the average BMI between the groups (23.7 vs. 39.3), and the percentage of deaths was significantly higher in the BMI < 30 group. The spirometry values were significantly lower in the BMI < 30 group. There were no significant differences in arterial gases or the hours of ventilator use, but there were significant differences in EPAP values (Table 4 and Fig. 3).

**Table 4:** Baseline variables and progression of COPD patients according to BMI (n = 176)

		COPD BMI < 30 kg/m^2^	COPD BMI ≥ 30 kg/m^2^	p value
n		62	114	
*Gender*				
Male	n (%)	33 (53.2)	60 (52.6)	
Female		29 (46.8)	54 (47.4)	
Age (years)	mean (SD)	66.5 (10.1)	63.3 (8.9)	0.0169
*Patient progression*				
Active patients	n (%)	34 (54.8)	94 (82.5)	0.0001
Dead		28 (45.2)	20 (17.5)	0.0001
*Lung function*				
FVC (%)	mean (SD)	56.2 (17.4)	60.8 (18.2)	0.0678
FEV_1_ (%)		26.5 (10.6)	41.8 (19.3)	0.0001
FEV_1_/FVC (%)		38.4 (11.6)	54 (15.3)	0.0001
*Arterial blood gas*				
p_a_O_2_ (mmHg)	mean (SD)	58.6 (13.2)	59.3 (10.8)	0.3886
p_a_CO_2_ (mmHg)		62.2 (10.7)	59.8 (8.4)	0.0700
HCO_3_- (mmol/L)		31.3 (6.3)	32.3 (5.4)	0.1976
Time in HMV (months)	mean (SD)	41 (31.9)	43.8 (31.8)	0.2921
*Compliance (h/day)*				
0–8	(%)	66.7	69.6	
> 8 to 16		30.9	30.4	0.432
> 16		2.4	0	
*Ventilator settings*				
IPAP	mean (SD)	16 (3.1)	17.1 (3.7)	0.0650
EPAP		7.1 (1.2)	7.7 (1.4)	0.0204
Backup respiratory rate		15.3 (2.4)	15.7 (2.6)	0.1941

*COPD* chronic obstructive pulmonary disease, *BMI* body mass index, *FVC* forced vital capacity, *FEV*_*1*_ forced expiratory volume in 1 s, *HMV* home mechanical ventilation, *IPAP* inspiratory positive airway pressure, *EPAP* expiratory positive airway pressure

### Health-related quality of life (HRQL)

**Fig. 4:**
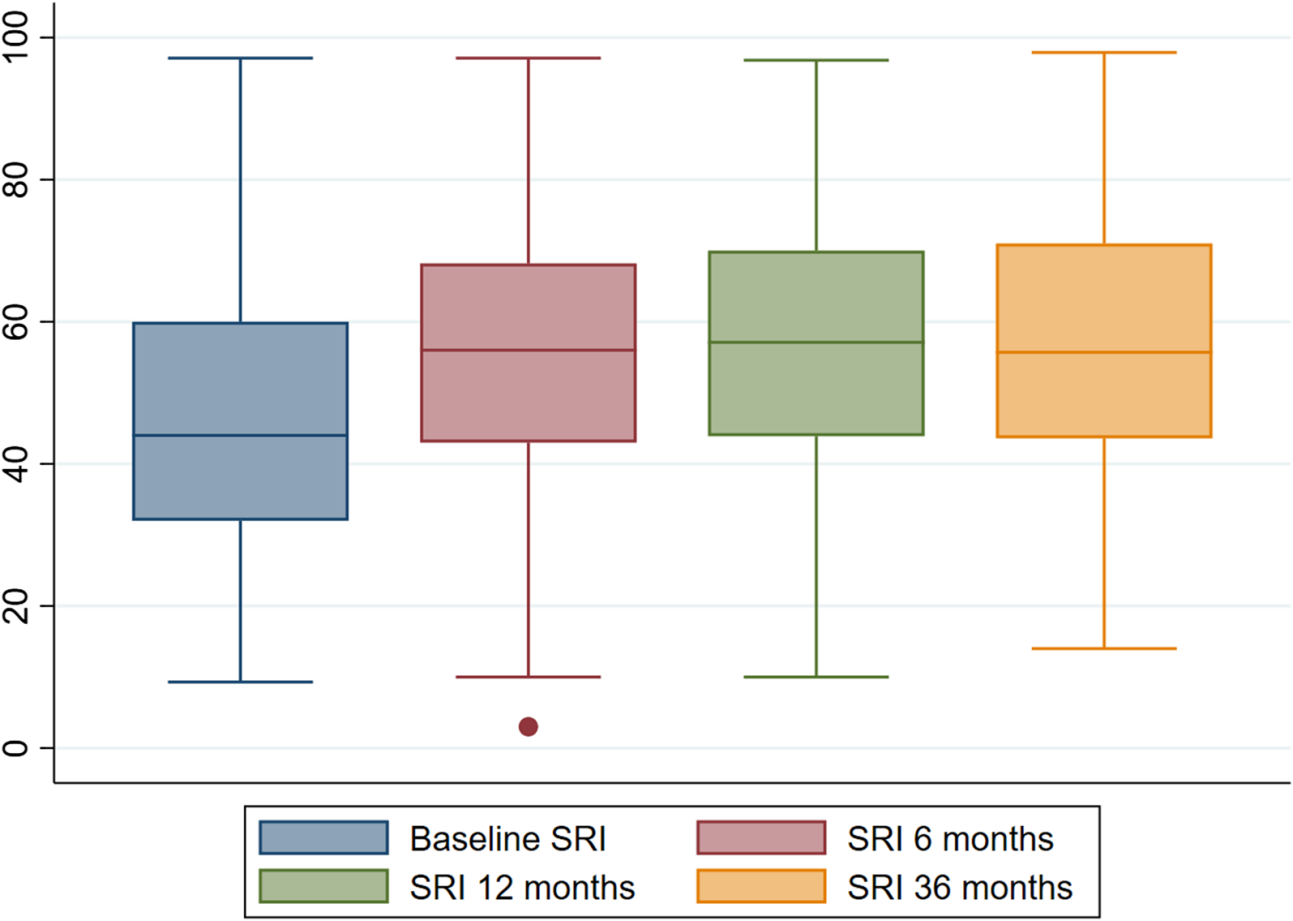
SRI score at 0, 6, 12 and 36 months in patients treated with HMV

The baseline SRI score for the whole population was 47 (± 17.9) points and showed a significant improvement at 6, 12 and 36 months [54.4 (± 17.2), 57.3 (± 17.3) and 57.1 (± 18.3) points, respectively, (*p* < 0.001)] (Fig. 4). The baseline SRI scores also differed significantly between active, discharged, and deceased patients [50.1 (± 19); 46.4 (± 19.6) and 43.2 (± 15.4), respectively (*p* = 0.001)].

### Causes of discharge and survival in the program

As of August 1, 2018, 675 patients were active in the program (61.1%), 329 were deceased (29.8%), and 101 patients (9.6%) had left the program. The reasons for leaving the program were poor adherence in 52 patients (49.5%), loss of data in 23 patients (21.9%), other modes of noncompliance in 4 patients (3.8%), voluntary withdrawal in 3 patients (2.86%), and improvement and discharge from the program in 23 patients (22.7%). The latter group included patients undergoing bariatric surgery, those with successful lung transplantation and those who were switched to continuous positive airway pressure (CPAP).

**Fig. 5:**
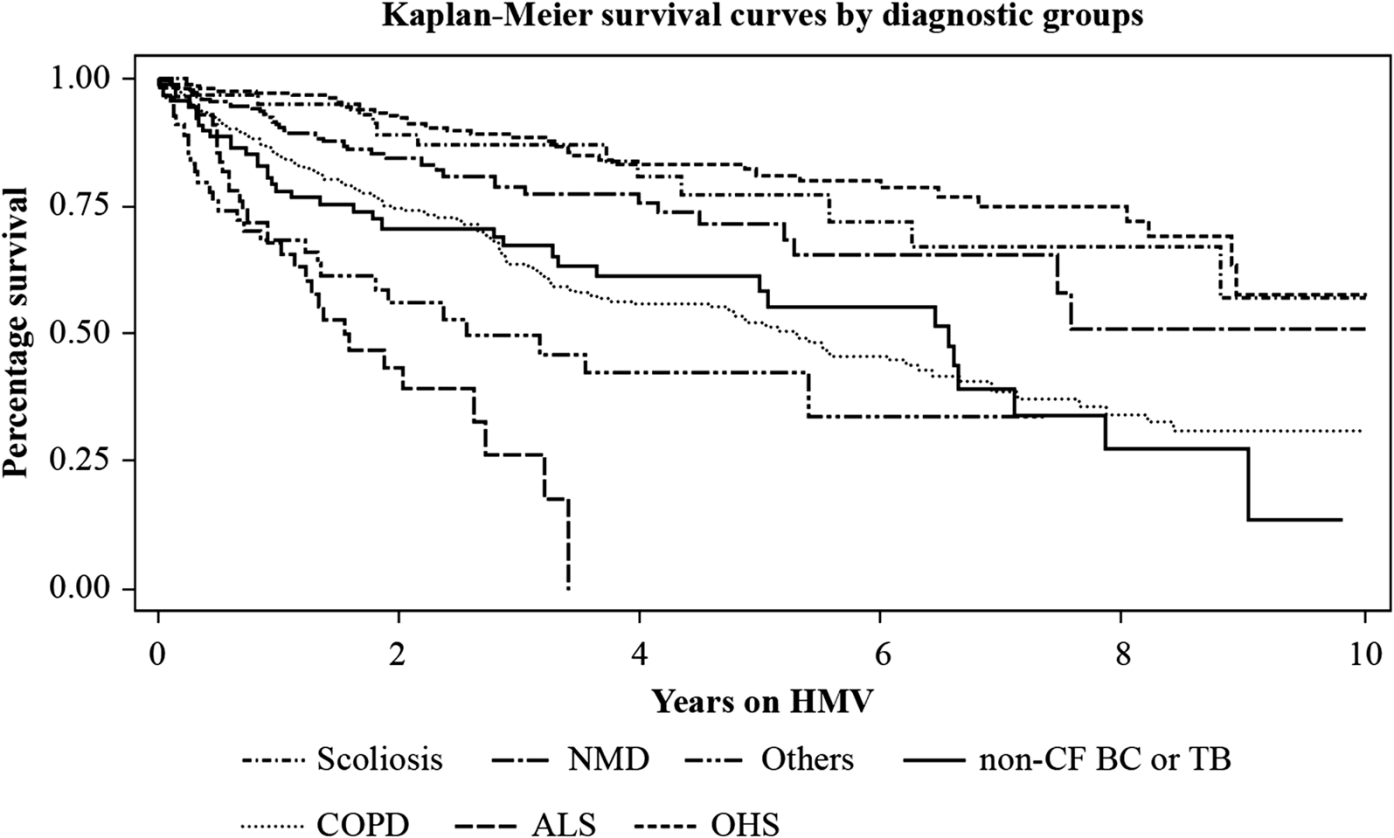
Survival time in years according to diagnostic groups

Kaplan–Meier analysis of survival for the whole population and for each group is shown in Fig. 5. As expected, the ALS group showed the lowest short-term survival (1- and 3-year survival of 67% and 26%, respectively). The groups with the lowest 5-year survival were COPD patients (52%) and non-CF BC or TB patients (58%). The longest 5-year and 9-year survival rates were observed in the OHS, scoliosis and NMD groups (81.2%, 77.4% and 71.4%, respectively, at 5 years and 57.7%, 57.2% and 50.9%, respectively, at 9 years).

In COPD patients with a BMI < 30 kg/m^2^, the percentage of deaths was significantly higher than that in those with a BMI ≥ 30 kg/m^2^ (Table 4 and Fig. 3).

## Discussion

Since the establishment of a home ventilation program in adults by the Chilean Health Authorities in 2008 and until 2017, 1105 patients were included in the register, with a mean of 110 patients per year. In 2017, there were 17.6 million inhabitants in Chile [22], 78% of whom depended on state health insurance coverage (FONASA for its acronym in Spanish) [12]. That same year, 722 adults and 701 children were enrolled in our national HMV programs [23]. These data allow us to estimate that the prevalence of HMV in Chile is 10.4 per 100 000 inhabitants. Interestingly, this prevalence rate is close to that estimated in the Eurovent Survey [6]

COPD and OHS were the most frequent diagnoses observed in our cohort, which is in line with data reported in other counties (see Table 5). Moreover, our data show a progressive increase in both pathologies as an HMV indication over the years (Fig. 2). This is consistent with data from the Geneva Lake Region for more than 20 years [9, 24].

**Table 5:** Published studies about HMV and the frequency of the different diagnostic groups

Author	Janssens JP	Windisch	Midgren/Laub	Garner	Povitz	Melloni	Schwartz	Cantero
Years	2003	2003	2007	2013	2017	2018	2020	2020
References	[24]	[19]	[25]	[26]	[9]	[8]	[27]	[10]
Area/Country	Switzerland	Germany	Sweden	Australia^a^	Canada	France	England	Switzerland
Type of study	cohort	cohort	cohort	survey	cohort	survey	cohort	cohort
Patients (n)	211	226	1526	2725	4670	4522	1210	489
Age (years)	63	57.3	58.6	57.5	58.5	70.3	65.1	71
p_a_CO_2_ (mmHg)	> 45	> 50	53.6	–	–	> 45	52.5	> 50
TIV (%)	excluded	excluded	6	3.1	7.7	…	12.4	excluded
NMD (%)	10	19	15	30	30.4		18	13
COPD (%)	27	34.5	16	8	18.8	6.3	24.5	39
OHS (%)	34	5.3	28	31	15.9	15.7	16.7	26
ALS (%)	3	27	11	8.4	7.5	1.2	21.6	3
Acute onset of ventilation (%)	–	–	26.6	–	23	–	–	45
Follow-up time (years)	7	3.25	10	4	12	15	10	3.3

*HMV* Home Mechanical Ventilation**,** *TIV* Tracheostomy invasive ventilation, *NMD* neuromuscular disease, *OHS* Obesity hypoventilation syndrome, *ALS* Amyotrophic lateral sclerosis

^a^New Zealand

The high rate of COPD and OHS patients on HMV could be explained by the high prevalence of smoking and obesity in Chile. Data from national health surveys in Chile [11] showed that the prevalence of smoking (daily or occasional) in 2003, 2010 and 2017 was 43.5%, 39.8% and 33.3%, respectively. Furthermore, the 2004 PLATINO survey established that the prevalence of COPD in Chile was 16.9% among individuals over 40 years of age [28]. The frequency of COPD in different cohorts varies between 6.3% up to 34.5% or 39% [8, 10, 19], similar to that of our cohort, which was 35%.

The reported prevalence of obesity in Chile increased from 23.2% in 2003 to 34.4% in 2017, and the expected number of mega-obese individuals (BMI > 40) increased from 148,000 people in 2003–415,000 in 2017 [11].

The median age of Chilean programmed people was 59 years, similar to published reports; however, Melloni et al. [8] and Cantero [10] reported a median age that exceeded 70 years (Table 5).

Schwartz et al. [27] and Laub and Midgren [25] described baseline PaCO2 levels of 52.5 and 53.6 mmHg, respectively, prior to the onset of HMV; in our cohort, the median baseline level was 58.2 mmHg, possibly due to the admission of patients with more severe disease or suboptimal therapeutic control. In addition, in our program, 72.6% of patients started HMV in a stable chronic condition, as reported by Povitz et al. [9] and Laub and Midgren [25], while in the Cantero cohort, only 55% of patients started HMV in this condition (Table 5).

The progressive increase over the years of ALS patients in our cohort is in line with increasing evidence of the effectiveness of HMV in this population. Indeed, HMV has become an essential part of the treatment of amyotrophic lateral sclerosis in recent years [29]. Other explanations could also account for this: the availability of more robust and sophisticated equipment allowing us to safely ensure long ventilatory support and the increasing expertise and skills of our group of physicians and physiotherapists in treating patients with a high requirement for ventilatory support.

In contrast to other series, the rate of patients ventilated through tracheostomy in our cohort was 5.2%. In other studies, it was between 6 and 12.4% (Table 5). This difference may reflect different practices among the countries. For example, in the Canadian cohort, the most frequent diagnosis was NMD (30.4%) [9], while the English cohort reported a diagnosis of ALS in 21.6% of patients [27].

The median SRI score at baseline in our cohort was 47 (35–62.1) points. This is lower than the score reported by Valko [30]. This discrepancy may be partially explained by differences in the socioeconomic status of the population included in both studies. The HMV Chilean program includes vulnerable patients with a low monthly income and low educational level [31]. They receive this benefit at no cost, financed by the national public health insurance system. When we compared the baseline overall SRI score between alive, deceased, and discharged patients, there were significant differences between the latter 2 groups compared to the first group, which may be related to a greater severity of the basic disease at the time of being postulated to enter the program. However, an analysis of the 7 dimensions that make up the score in each of the groups is necessary to identify those that generate the differences.

In 2020, Schwarz analysed the time elapsed from admission to death of 1210 patients on HMV in England and described that patients with ALS had the lowest mean survival, 7 months, whereas patients with OHS on HMV had the longest survival, 33 months. The Swedish group also described that the worst survival was observed in patients with ALS, with a 20% survival rate at 2 years and a 5% survival rate at 5 years [25]. In our cohort, the mean time on HMV in each group was slightly higher than that in the UK cohort (14 ± 10.4 months in patients with ALS and 42.3 ± 32.4 months in OHS patients). In our series, the patients with COPD with a BMI < 30 kg/m^2^ had a lower survival rate, were older and had worse functional outcomes than those with a BMI ≥ 30 kg/m^2^. It is possible that patients with a BMI ≥ 30 had overlap syndrome, but we did not have available basal polygraphs for all the patients (Table 4 and Fig. 3).

One novel aspect of our work is that we assess the implications of the familiar environment. We used the APGAR family dysfunction score, which evaluates the functionality of the family group. A score of higher than 7 suggests a highly functional family [16, 17]. In our cohort, this score was 10, suggesting important family support to the patient for the management of their disease.

The degree of severity of CRF in patients admitted to our program was higher than that in other series (Tables 3, 5). However, we strictly respect the criteria of the ACCP consensus conference [15]. One explanation for that is that among the criteria for admission to our program, it is established that "the patient must have been hospitalized for decompensation with CRF in the last 12 months". This condition was necessary at the time of the creation of the program to reduce the number and duration of hospitalizations of the most severe patients. A revision of admission criteria to the program is now in progress to soften that condition with the aim of allowing an earlier admission of less severe patients.

In our cohort, the high compliance with the use of NIV with an average hourly use of 7.3 h can be explained by the continuous education of the patients and caregivers, who were regularly visited at their home 1 to 3 times a week by a physiotherapist and once a month by a nurse, and the programmed pressure levels were selected to make the patients feel comfortable. In addition, according to the APGAR survey, the relatives gave the patients all the necessary support at home. All these factors contributed to compliance with NIV.

### Program weaknesses and strengths

Baseline functional data at program admission, such as maximum inspiratory pressure (MIP), lung volumes and capacities, carbon monoxide diffusing capacity (DLCO) and polygraphs, were not available for all patients because some hospitals where the patients were evaluated did not have the equipment to acquire these data. The measurement of DLCO and lung volumes and capacities has been described as having prognostic value, especially in COPD patients [32].

The SRI questionnaire was completed by all patients who had the ability to provide reliable information. The present cohort only represents the adult beneficiaries of the Chilean public health system, and it does not consider adults with private health insurance in need of HMV, whose number we do not know.

We did not have detailed information about patients who died while waiting to be evaluated and admitted to the program.

The strengths of this study include the fact that the HMV program was started gradually, first in the metropolitan region, which includes the capital of Chile, Santiago de Chile (6.1 million inhabitants); 3 years later, it was expanded to different regions of the country; 6 years later, patients who needed invasive ventilation were included. Additionally, there has been low turnover in the technical team responsible, which includes medical doctors, physiotherapists, and nurses as well as professionals in hospitals located in different regions of the country.

Another important issue is that our program is a centrally supervised national-based program, covering more than 75% of the population of the country, including a protocolized follow-up strategy and managed by a pluridisciplinary group including medical doctors, physiotherapists, and nurses as well as professionals in hospitals located in different regions of the country. This differs from other countries in which the model of care includes different actors, i.e., care is often provided by private or semi-public health care companies and/or community providers sometimes with different policies [33].

## Conclusion

The most frequent diagnoses in our cohort of 1105 patients were COPD, OHS and NMD. Patients with a low quality of life score at admission were more hypercapnic than those in similar series from other countries. The patients were socioeconomically vulnerable, were distributed throughout the country, adapted very well to the use of HMV, and had a time of stay in the program like that of other series. The HMV program offers continuity of home ventilatory support for individuals transferred from the children's national program. The best survival was observed in patients with OHS, scoliosis and NMD, and the number of patients who were discharged from the HMV program due to resolution of their underlying disease was small. SRI improved significantly in the total group at 6, 12 and 36 months.

## Supplementary Information

**Additional file 1**. Technical standard for HMV programs. Ministry of Health, versions 2008, 2012 and 2013, Chile.doc.

## Data Availability

All data generated or analysed during this study are included in this published article. The datasets generated and/or analysed during the current study are not publicly available due to the ethical standards established by the law of duties and rights of patients N° 20,584 promulgated in 2012 by the Chilean State but are available from the corresponding author on reasonable request. [HMV Chile DATABASES WITHOUT PATIENTS NAMES.xlsx].

## Abbreviations

ALS: Amyotrophic lateral sclerosis
APGAR: Screening for family dysfunction
BMI: Body mass index
COPD: Chronic obstructive pulmonary disease
CRF: Chronic respiratory failure
DLCO: Diffusion capacity of lung for carbon monoxide
EPAP: Expiratory positive airway pressure
FVC: Forced vital capacity
FEV_1_: Forced expiratory volume in 1s
HMV: Home mechanical ventilation
IPAP: Inspiratory positive airway pressure
IQR: Interquartile range
NMD: Neuromuscular disease
OHS: Obesity hypoventilation syndrome
p_a_CO_2_: Carbon dioxide arterial pressure;
non-CF BC or TB: Non-cystic fibrosis bronchiectasis or tuberculosis
