# Supplementary material for: Results of the home mechanical ventilation national program among adults in Chile between 2008 and 2017
Source: BMC Pulm Med. 2021 Dec 2;21:394. doi: 10.1186/s12890-021-01764-4 (PMC8641152; doi:10.1186/s12890-021-01764-4)
Supplement: Supplementary file 1 — Additional file 1. Technical standard for HMV programs. Ministry of Health, versions 2008, 2012 and 2013, Chile.doc. [file 12890_2021_1764_MOESM1_ESM.doc]

**Home Non-Invasive Ventilation Programme for**

**Adults in Primary Health Care –**

**AVNIA TECHNICAL STANDARD**

**May 2008**

First Edition: 18 June 2008

Second Edition: 19 January 2012

Third edition: 06 May 2013

- **Presentation 3**
- **Glossary of Abbreviations 4**
- **Introduction 5**
- **Programme Structure and Objectives 8**
- **Evaluation of Objectives 9**
- **NIV Definition 9**
- **NIV Selection Criteria 10**
- **NIV Inclusion Criteria 10**
- **NIV Exclusion Criteria 11**
- **Equipment and Types of Non-Invasive Ventilation 12**
- **Programme Context 13**
- **Staff Responsibilities 15**
- **Admission Procedure 20**
- **Admission Modes 21**
- **AVNIA team activity 21**
- **Physical Facilities 22**
- **Table 1. Admission activities 23**
- **Visiting Scheduling 25**
- **Emergency Plan 25**
- **Follow-up 26**
- **Goal achievement 27**
- **Education/Training 29**
- **Annex 1: Titration 30**
- **Annex 2: Cross-Consultation Request 31**
- **Annex 3: Functional assessment and Quality of Life Score**

**before starting NIV 34**

- **Annex 4: Informed Consent 35**
- **Annex 5: Equipment Loaning Contract 40**
- **Technical Group – Standards 46**
- **National and International References 47**

**PRESENTATION**

This document includes the technical standards designed for implementing a National Home Non-Invasive Ventilation Programme for Adults (AVNIA, for its acronym in Spanish) with chronic diurnal or nocturnal hypoventilation secondary to obstructive bronchial disease, neuromuscular diseases, or rib cage anomalies.

Solutions are provided so that the requirements of these patients are met by transferring technology, derived benefits, and training and supervision in the home by specialised regional teams of primary health care professionals.

The rationale and criteria for the distribution of resources associated with the programme are based on three components:

1.- Detection, evaluation, and referral 2.- Training

3.- Field supervision by regional primary health care teams

Until December 2011, the programme offered coverage only in the Metropolitan Region, where a total of 311 patients benefited, of which 200 remain active. Since January 2012, coverage expanded with regional care teams to Regions V, VI, and VIII to initiate the gradual admission of patients meeting the criteria for admission to the programme.

This programme considers it essential to establish the foundation of an organisational strategy integrating the different health care levels, but having as its central axis the home and family as direct agents of the actions, allowing successful non-invasive ventilation in patients with diurnal hypoventilation derived from various diseases, whether of the respiratory pump, airway, or lung parenchyma. The actions of the health team in this model of home non-invasive ventilation are considered facilitating of the self-care of patients and their families.

**GLOSSARY OF ABBREVIATIONS**

| ABGs | arterial blood gases |
| --- | --- |
| AVNIA | Home Non-Invasive Ventilation Programme for Adults |
| BIPAP | bi-level intermittent positive airway pressure |
| COPD | chronic obstructive pulmonary disease |
| CPAP | continuous positive airway pressure |
| DTC | diagnosis and treatment centre |
| EPAP | expiratory positive airway pressure |
| FRC | functional residual capacity |
| FVC | forced vital capacity |
| IC | inspiratory capacity |
| INT | National Thorax Institute |
| IPAP | inspiratory positive airway pressure |
| IRA | National Programme for Acute Respiratory Infections |
| MEP | maximal expiratory pressure |
| MIP | maximal inspiratory pressure |
| NC | PHC Nurse - AVNIA Coordinator |
| NIV | non-invasive ventilation |
| NRE | non-respiratory event |
| NU | PHC Nurse - AVNIA Team |
| OHS | obesity-hypoventilation syndrome |
| OSAHS | obstructive sleep apnoea/hypopnea syndrome |
| PHC | primary health care |
| PMC | Programme Medical Coordinator |
| PMSC | Programme Medical Sub-coordinator |
| PP | Programme Physician |
| PT | PHC Physical Therapist - AVNIA Team |
| PTC | PHC Physical Therapist - AVNIA Coordinator |
| PU | pulmonologist |
| QoL | quality of life |
| RE | respiratory event |
| RV | residual volume |
| SAMU | mobile emergency care service |
| SaO_2_ | pulse oximetry |
| SENADIS | National Disability Service (formerly FONADIS) |
| SRI questionnaire | Severe Respiratory Insufficiency questionnaire |

**INTRODUCTION**

In Chile, non-invasive ventilation (NIV) in adults has been conducted in the acute patient setting, typically in exacerbations of patients with decompensated COPD due to pneumonia, viral infections, pneumothorax, or pulmonary thromboembolism accompanied by severe respiratory failure with respiratory acidosis, requiring hospitalisation in the intermediate treatment unit (ITU)^1^ or intensive care unit (ICU).

Home NIV programmes in Europe establish that the prevalence of use of this ventilatory support varies from 2 per 100,000 inhabitants in Poland to 30 per 100,000 inhabitants in France. In 1980, ANTADIR (Association Fédérative Nationale pour le Traitement A Domicile de l'​Insuffisance Respiratoire chronique) had 11,000 home patients and by 2001, 120,000 patients, including those with stage IV COPD, neuromuscular diseases, and OSAHS^8^.

In patients with stable chronic COPD with global respiratory failure and CO_2_ retention, it has been shown that intermittent positive pressure applied to the upper airway via a nasal mask in minimum cycles of 3 hours a day increases FVC and IC, reducing hyperinflation and the work of breathing^2^. Likewise, using home NIV in patients with respiratory diseases associated with chronic diurnal and/or nocturnal hypoventilation improves the quality of life and reduces the costs of unscheduled hospitalisations and the consumption of hospital resources in critical care units^3^.

National experiences^4,5,6^ addressing the issue of NIV in children and home ventilation systems have shown the latter can efficiently be used to treat patients with chronic respiratory failure at home, reducing hospitalisations, promoting reintegration into social and school activities with few complications, and improving the quality of life with significant cost-effectiveness.

In 2007, the Chilean Society of Respiratory Diseases appointed a Commission to develop a Chilean Consensus on Non-Invasive Ventilation encompassing all related topics, which was published in 2008 in *Revista Chilena de Enfermedades Respiratorias* [Chilean Journal of Respiratory Diseases]^12^.

In May 2008, the public system created a Non-Invasive Ventilation Programme for Adults (AVNIA) for the systematic delivery of this type of home ventilation. In the private sector, a similar system is offered by some companies, at a much higher cost.

The patients receiving the benefit of diurnal and/or nocturnal NIV are those with stage IV COPD with home oxygen therapy, chronic respiratory failure, very high PaCO**_2_,** and repeated hospitalisations.

A recent meta-analysis concluded that:

1. Patients with more severe hypercapnia benefit more from NIV.
2. The benefits of NIV are greater in patients better trained in its use.
3. Long-term use of NIV achieves greater benefit.
4. The greater the difference between IPAP and EPAP (support pressure), the greater the benefit of NIV.
5. Home NIV supervision achieves greater success in this type of treatment^7^.

Restrictive patients, patients with neuromuscular diseases, and those with kyphoscoliosis currently have a longer life expectancy following diagnosis thanks to early NIV.

Obesity is another prevalent disease growing in Chile. The last National Health Survey of 2010 established that 28.4% of Chileans suffer from all obesity grades, predisposing them to certain diseases, including OSAHS and OHS. Of the 300,000 morbidly obese people in Chile according to the health survey, 150,000 (50%) will develop OSAHS, requiring treatment, and 30,000 (10%) will develop OHS. This number exceeds all HIV-positive patients already diagnosed and under treatment, plus all vascular diseases put together. According to Budweiser *et al*. (2007), the mortality rate of patients with OHS is 18% per year.

Sugerman *et al*. (1986) established that 38 (14%) of 263 severely obese patients who were to undergo gastric surgery for weight reduction had respiratory insufficiency (PaO_2_ <55 mmHg and PaCO_2_ >47 mmHg) and therefore suffered from OHS^9^. Kessler *et al*. (2001) showed all the alterations produced during sleep in severely obese patients, such as deep, repeated and prolonged desaturations, as well as the early development of pulmonary hypertension, eventually lead to a large demand for health care services^10^.

At the INT, an occupancy of 1500 bed-days/year is exclusively generated by 25 patients with chronic respiratory failure who lack home NIV equipment. Similar situations may occur in other hospitals. Considering this information, the Respiratory Health Unit of the Chilean Ministry of health (MINSAL, for its acronym in Spanish) proposed the implementation of a home NIV programme for adults, whose objective would be to deliver to patients meeting the admission criteria, modern, safe, supervised, and cost-effective home ventilation equipment.

**PROGRAMME STRUCTURE AND OBJECTIVES**

## GENERAL OBJECTIVES

- Reduce morbidity and mortality in patients older than 20 years, belonging to the public health system, suffering from neuromuscular disease, obesity-hypoventilation syndrome, or severe kyphoscoliosis.
- Contribute to improving the quality of life of patients with chronic alveolar hypoventilation.
- Reduce hospitalisation days per year in patients with chronic alveolar hypoventilation.
- Increase the efficiency of the health care network.

## SPECIFIC OBJECTIVES

- - Reduce symptoms and morbidity related to chronic diurnal and nocturnal hypoventilation.
  - Stabilise and reverse chronic nocturnal hypoventilation.
  - Reduce hyperinflation, its consequences, and the work of breathing in obstructive patients.
  - Avoid invasive ventilation/tracheostomy.
  - Restore functional independence and improve quality of life.
  - Strengthen the diagnostic, therapeutic, and management capacity of the health care network with an emphasis on PHC, in the field of home care.
  - Free up intrahospital resources in adult critical care units (ICU-intermediate), mainly in winter periods, allowing the admission of other patients with acute illnesses.
  - Deliver non-invasive ventilation equipment, implementing a flowchart of programmatic actions.
  - Transfer technology and training to PHC for the coordinated management of patients with special respiratory needs.
  - Optimise health system resources by managing these patients on an outpatient basis, with high cost-benefit ratio.

**EVALUATION OF OBJECTIVES**

- - Compare days of hospitalisation per year from 2 years before entering the programme and during its implementation.
  - Compare the results of the quality-of-life score before and during the implementation of the NIV programme.
  - Evaluate the functional assessment parameters of the disease: arterial blood gases, MIP-MEP, walk test, and spirometry before and during the implementation of the NIV programme.
  - Evaluate compliance and adherence to ventilatory support with the aid of the memory card and by assessing the hours of use of the equipment.

## DEFINITION

- - **Non-Invasive ventilation**: Non-Invasive ventilation is all ventilatory support provided to a patient using flow-generating equipment with positive airway pressure delivery by CPAP or BIPAP, through interfaces such as nasal, oronasal, or full-face masks. The programme provides patients with global respiratory failure with a BIPAP device, aimed at reducing the work of breathing and complementing ventilatory function.
  - **CPAP:** Continuous positive airway pressure.
  - **BIPAP:** Bi-level positive airway pressure.
  - **AVAPS:** Average volume-assured pressure support.
  - **Event:** Term denoting any deviation in the usual clinical behaviour of the patient. Designated as RE or NRE depending on whether it is related or not to ventilatory support.

## SELECTION CRITERIA FOR HOME NIV

- Disease requiring chronic non-invasive ventilation.
- Stable clinical condition.
- Type II Respiratory failure (hypercapnic) without respiratory acidosis.
- Oxygen requirements ≤4 l/m to maintain SpO_2_ ≥88% without BIPAP
- IPAP – EPAP not >26 and 10 cm H_2_O, respectively (in a hospitalised patient)
- Carer/family member or responsible adult, adherent and with social network support.
- Adequate housing with basic services and adequate facilities (electricity, telephone).
- Informed consent and equipment loan contract signed by the patient and/or carer/family member/responsible adult.

## INCLUSION CRITERIA

Patients with chronic alveolar hypoventilation secondary to the following diseases:

- Oxygen-dependent stage IV COPD, with more than 1 hospitalisation in the past 12 months and daytime PCO_2_ >55 mmHg or daytime PCO_2_ ≥50 mmHg associated with deep and numerous nocturnal desaturations.
- Overlap syndrome, COPD and OSAHS, with daytime PCO_2_ >50 mmHg.
- Tuberculosis sequelae with daytime PCO_2_ >50 mmHg.
- Neuromuscular disease with daytime PCO_2_ >45 mmHg.
- Chest wall disease (kyphoscoliosis, thoracoplasty, etc.) and daytime PCO_2_ >50 mmHg.
- Obesity-hypoventilation syndrome
- Pulmonary fibrosis or cystic fibrosis awaiting lung transplantation.
- NIV-dependent ICU or ITU patient.
- AVIA Programme patient who turns >20 years.

## EXCLUSION CRITERIA

- Not having a family support network and lacking basic housing conditions referred to in the inclusion criteria.
- Mask (interface) intolerance.
- Any type of cancer with a life expectancy <6 months.
- Cardiorespiratory arrest in the last 4 weeks.
- Rapidly progressive neuromuscular disease.
- Swallowing disorder with lack of glottic protection.
- Lack of respiratory autonomy with supplemental O_2_ outside of positive-pressure support.
- Need to use NIV >20 hours a day.
- Permanent requirement for tracheal suctioning.
- Tracheostomy patient.
- Severe organic brain damage confirmed by a neurologist.
- Active tobacco addiction, not stopped in >6 months or with risk of relapse despite treatment (valid for other drugs or alcohol).
- Decompensated and untreated psychiatric illness, i.e., schizophrenia.

## EQUIPMENT AND MODES OF NON-INVASIVE VENTILATION

- BIPAP in spontaneous/timed (S/T) mode: for a restrictive, obstructive disorder, or insufficient respiratory command.
- BIPAP with AVAPS system for patients with neuromuscular or rib cage disorders in whom an assured tidal volume is considered essential.
- Humidifier-heater base in patients who are particularly obstructive or use ventilatory support >12 hours a day.
- Accessories:
  - Nasal and oronasal mask interface.
  - Corrugated tubing.
  - Filters.
- Saturation monitor: for periodic nocturnal oximetry recording according to protocol.
- Respiratory polygraphy: for patients with suspected association with OSAHS (e.g., COPD or OHS patients).

## PROGRAMME CONTEXT

The home NIV programme will be developed mainly in two scenarios:

**Chronic admission mode** (Figure 2): Patient at home with an indication for home BIPAP. The PU must fill out the Electronic Consultation Form available on the programme's website ([www.avnia.cl,](http://www.avnia.cl/) user: inter/password: interconsulta-258). This information is received by programme staff. If with the information available online, the patient qualifies, they will be scheduled by telephone to be evaluated by the AVNIA PP at the polyclinic attached to the hospital closest to their residence. After the clinical evaluation and tests (ABGs, pulmonary function, radiological imaging, EKG), an evaluation by a social worker and mental health status assessment are added. If the patient meets the admission criteria, they will be scheduled to undergo NIV titration (see Annex 1).

Titration is performed with the patient hospitalised, depending on bed availability at the base hospital (otherwise, at the patient's home). Titration consists of a 48-hour scheduled and abbreviated hospitalisation, where pending baseline tests are completed on the first day and a baseline nocturnal oximetry is performed (if the patient is a home oxygen user, with the litres indicated by the ambulatory oxygen programme). On the second day, titration is performed according to the protocol, with the most suitable interface for the patient's face, determining the highest pressure tolerated by the patient. Once used to the mask, nocturnal oximetry is performed on the second night by BIPAP with the previously determined inspiratory and expiratory pressures (IPAP and EPAP, respectively).

Finally, the patient and/or family carer are trained in handling the equipment. Once the above is done, the patient will be sent home with the equipment, interfaces, heater, and backup batteries if the case warrants it.

The patient will receive a visit schedule corresponding to each AVNIA specialist and will periodically attend the AVNIA polyclinic closest to their residence, according to the follow-up protocol.

The patient will continue with their scheduled check-ups with the attending PU at the appropriate hospital or DTC or where they attend for follow-up. Additionally, the patient will maintain regular control of their different morbidities and receive required vaccines at their clinic of residence.

In this way, tertiary, secondary, and primary health care levels are integrated. Moreover, the necessary technology is transferred to manage these patients at home, a fundamental part of the PHC strategy.

In mild exacerbations, the patient will be evaluated in primary or secondary care by a PU as unscheduled visits; in moderate/severe exacerbations with clinical deterioration, respiratory distress, and evident increase in the patient's ventilatory requirements, an assessment will be performed in the emergency department (ED) or at home (SAMU). At the patient's home, ascertaining a stable clinical condition or an exacerbation will be the responsibility of the direct carer. The programme physical therapist (PT) and nurse (NU) must recognise the clinical situation of the patient at the time of evaluation at regularly scheduled visits.

**Acute admission (inpatient) mode** (Figure 3): Having controlled the initial severity of symptoms, patient who requires the use of intermittent diurnal and/or nocturnal BIPAP to maintain good ventilatory mechanics with no arterial acidosis. Furthermore, the patient needs 4 or fewer l/m of oxygen with or without BIPAP. This patient will be evaluated by one of the PPs, where a stable pH must be confirmed, and the only problem is PCO_2_ retention. The patient must have access to the family support network and habitability must be verified by the social worker of the health care network. A BIPAP kit will be given to the patient after training the health personnel of the unit responsible for the patient in the hospital. The most suitable interface is selected, programming the equipment with parameters similar to hospital equipment. Only nocturnal oximetry is performed with BIPAP equipment. After a minimum of 48 hours of equipment use, the discharge will be scheduled, and the family will be trained for home handling. Four weeks later, the patient is scheduled to attend an AVNIA polyclinic to evaluate adherence and tolerance to NIV equipment, with a possible one-week window without equipment and completion of the chronic admission flowchart (Figure 1).

## STAFF RESPONSIBILITIES

The responsibilities assigned to each team member are described below (Figure 4).

## PULMONOLOGIST COORDINATOR (PC)

- Manage the home NIV programme.
- Chair monthly meetings with the programme's team members.
- Advise and audit clinical cases.
- Oversee programme equipment, maintenance and annual expansion of equipment, accessories and complementary diagnostic equipment.
- Participate in weekly meetings in the MINSAL Respiratory Health Unit for accountability.
- Evaluate programme process and outcome indicators.
- Organise, promote and oversee the training and coaching of team members.

## PULMONOLOGIST SUB-COORDINATOR (PSC)

- Assist with the responsibilities of the programme coordinator.
- Authorise the admission of potential patients, until regional teams are trained.
- Participate in weekly meetings in the MINSAL Respiratory Health Unit for accountability.
- Organise monthly meetings with the programme's team members.
- Collect and evaluating information on programme process and outcome indicators.
- Organise, promote, and oversee the training and coaching of team members.

## PROGRAMME PHYSICIAN (PP)

- Evaluate, approve or reject cross-consultations (inpatient visit or polyclinic appointment).
- Authorise patient admission.
- Available to advise the NU or PT on treatment questions by telephone.
- Responsible for overseeing ventilatory support and responsible for parameter adjustments, equipment or mask changes together with the PT or NU.
- Attend the AVNIA polyclinic attached to a hospital weekly for patient admission evaluation and controls according to follow-up protocol.

## NURSE COORDINATOR (NC)

- Coordinate patient distribution to the NU Team.
- Coordinate the PP polyclinic for evaluation of patients awaiting admission and controls of patients active in the programme.
- Oversee and evaluate compliance with admissions records, surveys, visits, REs and NREs on the programme's website by PP, PT and NU.
- Oversee the equipment maintenance plan delivered by the company awarded the tender.
- Order and distribute equipment (history)
- Conduct joint home visits with PT and NU with patients with special needs
- Coordinate and participate in monthly meetings with the AVNIA Team.
- Participate in weekly meetings at the MINSAL Respiratory Health Unit
- Coordinate training for and with AVNIA team members.
- Organise the updating of the User's Manual with the participation of the entire AVNIA Team.
- Coordinate vacations, time off and/or leaves of absence. Reassign patients to the NU Team.
- Assign a replacement in case of absence.

## NURSE (NU)

- Make the first home visit during the first week of admission to the programme.
- Make monthly follow-up visits.
- Perform arterial blood gas tests, obtain the QoL score by applying the SRI questionnaire and apply the family Apgar scale upon admission, and then according to protocol. Apply the Goldberg Depression Scale.
- Record visits on a web page and home care notebook.
- Web records and Excel support: QoL score, Apgar, ABGs and hospitalisations.
- Apply the user's satisfaction survey.
- Check treatment adherence and compliance with indications, identify exacerbations.
- Initiate reports on REs and NREs, requesting advice from the PP, activating a contingency plan.
- Perform and strengthen continuing education/training of the patient and/or family carer.
- Participate in monthly meetings of the AVNIA Team.
- Maintain regular communication with the Clinic Team closest to the patient's residence (the medical office responsible for the patient).
- Assist in the medical polyclinic, with patient scheduling, records, etc.

## PHYSICAL THERAPIST COORDINATOR (PTC)

- Coordinate patient distribution to the PT Team.
- Coordinate PT assignment for training in using the NIV equipment of patients.
- Coordinate vacations, time off and/or leaves of absence.
- Reassign patients to the PT Team.
- Assigning a replacement in case of absence.

## PHYSICAL THERAPIST (PT)

- Make first home visit during the first week of admission to the Programme.
- Make visits once a week to check equipment and specific protocolised muscle training. After 3 months, if the patient maintains clinical and ventilatory stability, reduce visits to 2 times a month. Except for patients with a tracheostomy who will maintain the weekly visit.
- Record visits on a Web page and home care notebook.
- Detect equipment failures and damage of accessories and give a prompt solution to these problems.
- Evaluate adherence to the team through the ENCORE card and sending it to the physician according to protocol.
- Perform functional tests upon admission and periodically according to the follow-up protocol.
- Initiate reports on RE and NRE, requesting advice from the PP, activating a contingency plan.
- Strengthen continuing education/training of the patient and/or family carer.
- Check the capacity of the patient and/or family carer, besides maintaining basic services providing security for programme implementation.
- Biannual nocturnal oximetry, performing equipment installation and removal, downloading and sending a digital report to the physician according to protocol.
- Web records and Excel support: functional exams, equipment programming, adherence and oximetry.
- Participate in the monthly meetings of the AVNIA Team.

## PRIMARY CARE PHYSICIAN (PCP)

- Unscheduled medical visit in mild exacerbations.
- Quarterly visit at the Primary Health Care Service. Preferably an acute respiratory disease physician.
- Manage health supervision, control of chronic non-respiratory diseases, immunisations, feeding, evaluation of the respiratory condition and recognition of occasional deterioration. Preferably an acute respiratory disease physician.
- Preparation of a biannual report to the responsible PU. Preferably an acute respiratory disease physician.

## EMERGENCY PHYSICIAN (EP)

- Evaluate the patient for moderate to severe exacerbations in the emergency department. The event must be communicated within 24 hours to the AVNIA team that will visit the patient and to the PP for recording.

## SAMU EMERGENCY TEAM

- Evaluate the patient during the emergency at home. The event must be communicated within the first hour of care to the PT and/or NU for recording and coordination according to the need for hospitalisation in the ICU/ITU, activating the contingency plan; hospitalisation will be prioritised in the centre where the patient is usually controlled or was last hospitalised.

## PULMONOLOGIST (PU)

- Manage patient admission, fill out the form on avnia.cl.
- Coordinate patient referrals and screening according to selection criteria.
- Propose initial ventilatory treatment.
- Available for medical evaluation at 24 to 48 hours post-exacerbations (DTC or hospital)
- Responsible for the biannual evaluation of the patient's condition and its reporting to the PP.
- Responsible for engaging the multidisciplinary team (cross-consultations).

## NEUROLOGIST

- Define specific diagnosis and location.
- Establish prognosis and speed of progression: stable, slow or rapidly progressive (communicate this to the family).
- Early consultation with pulmonologist on suspicion of nocturnal hypoventilation.
- Fill out form for Disability Certificate (SENADIS).

## OTHER SPECIALISTS

**-PHYSICAL THERAPIST DTC** (DTC or hospital): Respiratory muscle training

**-GASTROINTESTINAL SURGEON:** Need for gastrostomy, bariatric surgery.

**ADMISSION PROTOCOL**

Once the patient is selected, the programme's admission protocol is started, which must include:

- Evaluation by the programme's multidisciplinary team: physician, physical therapist, and neurologist.
- Visit by the social worker to evaluate home conditions, the feasibility of carrying out a programme based on the characteristics of family collaboration.
- Mental health assessment at the local mental health clinic.
- Signing of informed consent form and loan contract for equipment delivery.

**COMPLEMENTARY STUDY FOR PATIENT SELECTION**

Pulmonary function (if the clinical condition allows it)

- - Spirometry without a bronchodilator.
  - MIP, MEP.
  - 6-minute walk test.

Arterial blood gases (ABGs)

- - Chronic Mode: pre-titration baseline ABGs, a test performed up to 3 months before titration is considered valid if it is not an exacerbation.
  - In-Patient Acute Mode: recent ABGs showing stable pH and taken with the litres of oxygen indicated by the home oxygen programme and <4 litres.
  - ABGs follow-up: at the 1st month, 3rd month, and every 6 months until 24 months, then once a year.

Nocturnal oximetry

- - Outpatient: first-night baseline oximetry and next night with BIPAP equipment, at the physical therapist's visit (first week after entering the programme).
  - Inpatient: baseline oximetry and next night with BIPAP equipment, at the physical therapist's hospital visit.

Postero-anterior and lateral chest X-ray

- - In all patients, those taken 6 months before admission evaluation are valid if the clinical condition is stable.

Computed axial tomography

- - At least 1 examination within the last 2 years in any former smoker, patient with bronchiectasis, or interstitial disease.

Respiratory polygraphy: a complementary study of suspected OSAHS associated with COPD or OHS.

Polysomnography: Selected cases.

Volumes and capacities: Specific cases.

Flexible fibreoptic bronchoscopy: Specific cases.

**SPECIFIC ADMISSION ACTION**

**AVNIA Team Activities**

**Chronic (outpatient or inpatient)**

- PMC accepts candidate
- PMC or PP perform evaluation, request complementary tests; select equipment, **Configuration and parameters**,
- Baseline nocturnal oximetry (with oxygen if user) and nocturnal oximetry with equipment.
- Training of patients and carers in the management and care of the equipment by the PT and NU.

**Acute (inpatient)**

- PMC accepts candidate
- PMC or PP visit the patient; perform evaluation, request complimentary tests; select equipment, configuration, and parameters.
- Delivery of equipment to the Unit, signing of the equipment loan contract by the Head of the Unit.
- Nocturnal oximetry with the equipment indicated for home use.

**Before starting home NIV**

- Training the patient and/or family member or carer in the use and care of the equipment and accessories
- Training the patient and/or family member or carer to recognise respiratory and non-respiratory exacerbations.
- Patient or family member and/or carer: sign a legal agreement to care for the equipment and return it (Loaning Contract) to the programme providers after completing the requirement at home (3 copies for the AVNIA programme). Sign informed consent form (2 copies, one for the patient and one for AVNIA)
- Emergency plan training
- QoL score and family Apgar scale.
- An information folder is provided:

-Medical history summary and emergency phone numbers.

-Emergency plan.

-Delivery of the equipment operation manual.

## PHYSICAL FACILITIES

- Programme coordination is located at the MINSAL Respiratory Health Unit, where the Ministry representatives and coordinators of the different levels of programme professionals meet weekly.
- Meetings are held monthly at the National Thorax Institute for the Metropolitan Region and in hospitals of the respective regional teams, to discuss clinical cases and provide information on administrative processes.
- The equipment is handled in the Metropolitan Region in the first instance. The AVNIA programme equipment warehouse is located at the National Thorax Institute, 5th floor north. Its internal movement is reported every 2 months to hospital management. Warehouse management is the responsibility of the Physical Therapist Coordinator and/or Sub-coordinator.

Table 1. Admission activities

| **ACTIVITIES** | **RESPONSIBLE** | **IN-PATIENT CHRONIC** | **IN-PATIENT ACUTE** | **OUTPATIENT CHRONIC** |
| --- | --- | --- | --- | --- |
| MEDICAL EVALUATION CHECKING ADMISSION CRITERIA | AVNIA PHYSICIAN | **YES**  **(IN-HOSPITAL EVALUATION)** | **YES**  **(IN-HOSPITAL EVALUATION)** | **YES**  **(EVALUATION IN AVNIA POLYCLINIC)** |
| HABITABILITY REPORT (DESIRABLE) | HOSPITAL  (Ideally on pre-admission) | **YES** | **YES** | **YES** |
| AVNIA ADMISSION DOCUMENTATION DELIVERY  (only for reading by the patient and/or family) | AVNIA | **YES** | **YES** | **YES** |
| BASELINE ABGs (without nocturnal NIMV): with O_2_ in dependent patients. | AVNIA HOSPITAL OR NURSE | **YES** | **YES** | **YES** |
| SPIROMETRY WITH AND WITHOUT BRONCHODILATOR | HOSPITAL | **YES** | **IN THE 4TH WEEK POST ONE WEEK WITHOUT NIMV** | **YES** |
| LAST MONTH PA AND LAT CHEST X-RAY | HOSPITAL | **YES** | **YES** | **YES** |
| EKG | HOSPITAL | **YES** | **YES** | **YES** |
| WALK TEST, MIP AND MEP | HOSPITAL OR KINESIOL. AVNIA | **YES** | **IN THE 4TH WEEK POST ONE WEEK WITHOUT NIMV** | **YES** |
| PSYCHIATRIC EVALUATION  (ideally) | HOSPITAL | **YES** | **WITH CROSS-CONSULTATION AT DISCHARGE** | **WITH PREVIOUS EVALUATION OR CROSS-CONSULTATION (according to the criteria of**  **AVNIA physician)** |
| NUTRITION  CROSS-CONSULTATION  (IF APPLICABLE) | AVNIA PHYSICIAN | **YES** | **WITH CROSS-CONSULATION AT DISCHARGE** | **YES** |
| NOCTURNAL BASELINE OXIMETRY AND/OR POLYGRAPHY, with O_2_ in dependent patients. | AVNIA | **YES** | **IN THE 4TH WEEK POST ONE WEEK WITHOUT NIMV AT HOME** | **YES** |
| BIPAP TITRATION | AVNIA | **YES** | **YES** | **YES (can be performed at home)** |
| TRAINING AND EDUCATION FOR PATIENT AND RESPONSIBLE FAMILY MEMBER | AVNIA | **YES** | **YES** | **YES** |

| NOCTURNAL POLYGRAPHY OR OXIMETRY WITH BIPAP PLUS O_2_  (O_2_-dependent pts). UP TO  ONE WEEK AFTER DISCHARGE | AVNIA | **YES** | **IN THE 4TH WEEK POST A WEEK WITHOUT NIMV AT HOME** | **YES (can be performed at home)** |
| --- | --- | --- | --- | --- |
| INFORMED CONSENT SIGNING: SIGNATURE OF PATIENT AND  responsible family member. 2 copies, one for the patient and another for AVNIA. | AVNIA | **YES** | **YES** | **YES** |
| BAILMENT CONTRACT SIGNING:  Bailee signature: can be the pt. or a responsible fam. member; 3 copies for AVNIA. | AVNIA | **YES** | **YES** | **YES** |
| QUALITY OF LIFE SCORE AND FAMILY APGAR SCALE | AVNIA NURSE | **YES** | **YES** | **YES** |
| WEEKLY HOME VISIT | AVNIA PHYSICAL THERAPIST | **YES** | **YES** | **YES** |
| MONTHLY HOME VISIT | AVNIA NURSE | **YES** | **ONE VISIT BEFORE THE 1st AVNIA MEDICAL CONTROL** | **YES** |
| MEDICAL CONTROL AFTER ADMISSION TO AVNIA | AVNIA PHYSICIAN | **1st, 3rd MONTH AND EVERY 6 MONTHS** | **AT ONE MONTH OF ADMISSION POST ONE WEEK PERIOD WITHOUT NIMV TO DECIDE CONTINUITY IN AVNIA** | **1st, 3rd MONTH AND EVERY 6 MONTHS** |

**VISIT** **SCHEDULING**

PT visits: once a week. After 3 months, if the patient maintains clinical and ventilatory stability, reduce visits to 2 times a month. Except for patients with a tracheostomy who will maintain the weekly visit.

- NU visits: 1 monthly visit.

-Visits to the PP: for outpatient admission evaluation, at the 1^st^ month of admission, at the 3^rd^ month, 6^th^ month, and every 6 months. Visit the PP with tests according to follow-up (Figure 5 and 6). Upon admission, 6th month, 12^th^ month, 18^th^ month, 24^th^ month. Next, controls with tests once per year

- Quarterly visits to PU and then biannual.

## EMERGENCY PLAN

In case of an event, the carers will be responsible for raising the alarm with a pre-designed guide for each patient.

The events will be catalogued according to present coding.

**RED:** Generates an emergency call to SAMU and ED consultation

Severe shortness of breath, SpO_2_ <90%, sensory involvement, cyanosis, bradycardia (individually defined).

**YELLOW:** Generates a call to the PT for telephone evaluation within 2 hours

Tachypnoea, isolated retraction, fever, increased secretions. If not resolved, the SAMU is called.

**GREEN:** Generates a call to PT or NU within 24 hours. General morbidity.

**FOLLOW-UP (Figures 1 and 2)**

**Scheduled monitoring activities**

Follow-up times are counted from the delivery of equipment to the patient. Medical check-ups at 1 and 3 months of admission aim to evaluate tolerance and adherence to therapy, which includes parameter adjustment, revision of interface leaks and humidification. It is complemented in this period and every 6 months with:

- - ENCORE pro SMARTCARD downloading
  - Nocturnal oximetry: continuous nocturnal record; minimum of 8 hrs.
  - ABGs

Furthermore, functional examinations, recording of anthropometric data, ventilator parameters, and days of hospitalisation are performed every 6 months. The severe respiratory insufficiency quality of life score (SRI), designed for patients in the home NIV programme, and the family Apgar scale are performed.

Figure 1. Follow-up guideline

# **Follow-up: Visit to the polyclinic PP**

**Adherence:**

- - - - Subjective: Patient
      - Objective: Encore Card

1st and 3rd month

- - - - **Tolerance:** Interfaces
      - Pressures
      - Nocturnal oximetry during

NIV

Main time Parameter adjustment

- - - **ABGs**

Figure 2. Follow-up guideline

**Follow-up: then, every 6 months**

Requesting exams

6th month, 12th month, 18th month,

24^th^ month.

Then, once a year.

- - - - **Baseline and post B2 spirometry MIP-MEP**
      - **6-min walk test**
      - **ABGs**
      - **Chest X-ray**
      - **Nocturnal oximetry during NIV**
      - **Check adherence-tolerance at each visit.**

## GOAL ACHIEVEMENT VERIFICATION

The programme establishes biannual goal achievement checks for the overall programme, for the benefits completed for each patient and the development of telephone achievement surveys.

1. Programme Goal achievement verification establishes measuring, at 6 months of activities, the number of patients evaluated to enter the programme in that period (Source: website), patients admitted to the programme, and the absolute number of current patients under treatment in the programme at the time of biannual evaluation.
2. The control of home care services delivered to patients includes recording the visits made in the electronic file, and each weekly physical therapist visit and monthly nurse visit in the home care notebook, including the printed record of arterial gases delivered by the portable gas laboratory.
3. Patients from the Metropolitan Region attend a biannual check-up at the National Thorax Institute, Hospital San José, Hospital San Juan de Dios, and Hospital Sotero del Río, where a pulmonologist from the programme evaluates the patient's laboratory tests and analyses the home care notebook of each patient to confirm the visits of the rest of the health team professionals. This information is summarised in an Excel spreadsheet expressly prepared freely allowing to verify and analyse all the data. The information is delivered to the MINSAL Respiratory Health Unit.
4. The average days of hospitalisation per year for each patient before entering the programme (2 previous years) are recorded and compared with the number of days of hospitalisation per year while in the programme. This allows demonstrating the significant reduction in the number of days of hospitalisation adjusted at 6 months and 1 year.
5. Adherence to therapy is recorded, and then retrieved from the memory card included in the equipment, providing us with the average hours of use in 24 hours. This information is obtained at 1, 3, 6, and then every 6 months. The patient is considered adherent to therapy if they use the equipment a minimum of 4 hours a day for >5 days a week. If after 1 month, the patient does not comply with the above, the equipment parameters, interface, and humidifier are checked, and education is reinforced. If after 3 months the patient does not meet minimum adherence, the equipment is removed, and non-compliance is recorded in the clinical file.

## EDUCATION/TRAINING

**Professional Education Protocol**

The overall content of the training programme for Physical therapists, Nurses and Physicians is as follows:

- Normal and positive-pressure ventilation
- Effects of positive pressure on different systems
- Non-Invasive Mechanical Ventilation: Terminology
- Pressure/time and flow/time curves in Non-Invasive Ventilation (NIV)
- Equipment for Non-Invasive Ventilation (NIV)
- Interfaces for Non-Invasive Ventilation (NIV). Selection and implementation
- Detection and resolution of problems in patients and equipment.
- Indications for Non-Invasive Ventilation (NIV) in different clinical situations
- A comprehensive evaluation of the patient requiring Non-Invasive Ventilation (NIV)
- Clinical management of the patient on Non-Invasive Ventilation (NIV)

**Education and training plan for patients/carers (initial and periodic reinforcement)**

- - Recognition of symptoms and signs of respiratory distress.
  - Recognition of decompensations in the underlying disease and other comorbidities (neurological, cardiological, etc.).
  - General information about the equipment to be used.
  - Care of equipment and accessories.
  - Oximetry and/or polygraph monitor (during use for study or control at home).

**Annex 1: AVNIA PATIENT TITRATION**

1.- Review of medical records.

2.- Selection of BIPAP equipment according to disease:

-COPD, OHS: Harmony

-Neuromuscular: Synchrony.

3.- Selection of interface and accessories (corrugated tubing, O2 connector, exhalation valve).

4.- Connection of patients to NIMV:

- If the patient is an NIMV user: Connect with the usual parameters and increase IPAP 2cm H_2_O every half hour up to the maximum tolerated by the patient.
- If the patient is NOT an NIMV user: Connect according to the suggested scheme up to the maximum tolerated by the patient.
- Then, at home, after an adaptation period, titration can be continued.

5.- In neuromuscular patients or those with rib cage anomalies, AVAPS/BIPAP Synchrony should be used, according to the scheme.

| **Schedule (time)** | **Parameters to check** | **COPD** | **OHS** | **Neuromuscular with AVAPS** |
| --- | --- | --- | --- | --- |
| **0 min.**  **14:30 hrs.** | **SaO_2_-HR-RR** | **IPAP= 8**  **EPAP= 4** | **IPAP= 8**  **EPAP= 4** | **IPAP=8-14 EPAP=4 Vt=8 ml/kg** |
| **30 min**  **15:00 hrs.** | **SaO_2_-HR-RR** | **IPAP= 10**  **EPAP= 5** | **IPAP= 10**  **EPAP= 5** | **IPAP= 8- 14**  **EPAP= 4** |
| **60 min**  **15:30 hrs.** | **SaO_2_-HR-RR**  **interface device** | **IPAP= 12**  **EPAP= 6** | **IPAP= 12**  **EPAP= 6** | **IPAP= 10- 16**  **EPAP= 5 Control Vt** |
| **90 min**  **16:00 hrs.** | **SaO_2_-HR-RR** | **IPAP= 14**  **EPAP= 7** | **IPAP= 14**  **EPAP= 7** | **IPAP= 10- 16**  **EPAP= 5** |
| **120 min**  **16:30 hrs.** | **SaO_2_-HR-RR**  **interface device** | **IPAP= 16**  **EPAP= 8** | **IPAP= 16**  **EPAP= 8** | **IPAP= 12- 18**  **EPAP= 6 Control Vt** |
| **150 min**  **17:00 hrs.** | **SaO_2_-HR-RR** | **IPAP= 18**  **EPAP= 8** | **IPAP= 18**  **EPAP= 8** | **IPAP= 12- 18**  **EPAP= 6** |
| **180 min**  **17:30 hrs.** | **SaO_2_-HR-RR**  **interface device** | **IPAP= 20**  **EPAP= 8** | **IPAP= 20**  **EPAP= 8** | **IPAP= 14- 20**  **EPAP= 6 Control Vt** |

**Annex 2: INTERCONSULTATION FOR AVNIA PRE-ADMISSION EVALUATION**

1. INTERCONSULTATION N° (AVNIA-IC001) (AVNIA USE):
   1. INTERCONSULTATION DATE:<dd/mm/aa> (automatic)
2. PATERNAL SURNAME:
   1. MATERNAL SURNAME: 2.2 NAMES:

2.3 SEX (M/F): <A> 2.4 DATE OF BIRTH:<dd/mm/aa> 2.5 AGE: ##

1. TAX ID# <A > 3.1 INSURANCE (FONASA 1, ISAPRE 2, UNINSURED 3): <A>
   1. REFERRING HOSPITAL (SOTERO DEL RIO 1, P HURTADO 2, L TIZNE 3, THORAX 4, OTHER 5)
   2. REGISTERED OFFICE OR RHS:
   3. RESIDENCE ADDRESS (STREET N°):
   4. TOWN OR VILLA:
   5. COMMUNE:
   6. TELEPHONE LANDLINE: 4.4 CELLULAR:

5.0 TYPE OF PATIENT (CHRONIC 1, ACUTE IN-PATIENT 2, AVNI 3, OTHER 4): _

6.0 USING HOME O_2_: 10.3 SINCE WHEN (MONTHS):

LITRES PER MINUTES: HOURS PER DAY

7.0 OXYGEN: FUNDING SOURCE (1 Hospital, 2 MUNICIPALITY, 3 Fonasa)

**8.0.*DIAGNOSTICS***

**IS THE PATIENT HOSPITALISED (YES OR NO) (IF THE ANSWER IS YES THE DIAGNOSIS IS DISPLAYED REASON FOR HOSPITALISATION)**

***ILLNESS OR CAUSE OF DECOMPENSATION***

PULMONARY EMBOLISM TERMINATION OF O_2_

COMMUNITY PNEUMONIA TERMINATION OF DRUGS

INFLUENZA OR PARAINFLUENZA ACUTE PULMONARY OEDEMA

ACUTE BRONCHITIS ACUTE CORONARY SYNDROME PNEUMOTHORAX ANOTHER DIAGNOSIS

OTHER

DIAGNOSIS………………………………………………………………………………………………………

**(IF THE ANSWER IS NO, IT SHOWS DIAGNOSIS OF UNDERLYING DISEASE)**

***9.0 DIAGNOSIS (MINIMUM 2) OF UNDERLYING DISEASE:<A >***

**UNDERLYING DISEASE**

***OBSTRUCTIVE DISEASES NEUROMUSCULAR RESTRICTIVE DISEASE***

COPD DUCHENNE MUSCULAR DYSTROPHY (NM1)

COPD plus OSAHS (Overlap) (COPD + OSAHS) STEINER DISEASE (NM2) ASTHMA stage LCFA (ASTHMA) BECKER MUSCULAR DYSTROPHY (NM3)

CYSTIC FIBROSIS BRONCHIECTASIS (CFB) SPINAL MUSCULAR ATROPHY (NM4)

NON-CYSTIC FIBROSIS BRONCHIECTASIS (NCFB) CHARCOT MARIE TOOTH DISEASE (NM5) SEQUELAE (TB) GUILLAIN BARRE DISEASE (NM6)

OTHERS: specify

***CHEST WALL RESTRICTIVE DISEASES ADDITIONAL AIRWAY DISEASE***

OBESITY-HYPOVENTILATION SYNDROME (OHS) TRACHEOMALACIA OR BRONCHOMALACIA SEVERE KYPHOSCOLIOSIS (KS) SEVERE OSAHS + CO_2_ RETENTION

DIAPHRAGMATIC HERNIA (DH)

UNI OR BILAT DIAPHRAGMATIC PARALYSIS (DP)

**PULMONARY RESTRICTIVE DISEASE**

ADVANCED PULMONARY FIBROSIS (PF)

***OTHERS:*** PNEUMOCONIOSIS

***COMORBIDITIES (MINIMUM THE THREE MOST IMPORTANT)***

TYPE-2 DIABETES MELLITUS (NIR) CANCER IN TREATMENT (BREAST CA)

TYPE-2 DIABETES MELLITUS (IR) LEUKAEMIA, COLON CA

HBP CORONARY CARDIOPATHY WITHOUT BYPASS GASTROSTOMY CORONARY CARDIOPATHY WITH BYPASS

COLOSTOMY ANOTHER IMPORTANT

**ARTERIAL BLOOD GASES PRE-INTERCONSULTATION:**

FIO_2_: PaO_2_: PaCO_2_: pH: BICARBONATE (ST)

***CARDIORESPIRATORY ARREST, ADVANCED NEUROLOGICAL DISEASE, OR CANCER***

15.0. PATIENT HAS ANY NEOPLASTIC DISEASE (YES OR NO):

15.1 TYPE OF DISEASE:

16.0 PATIENT NEEDED CPR IN THE LAST 4 WEEKS (YES OR NO):

17.0 PATIENT HAS ALZHEIMER'S DISEASE (YES OR NO):

18.0 PATIENT SUFFERS FROM MULTI-INFARCT DISEASE OR SEVERE NEUROLOGICAL SEQUELAE (YES OR NO)

19.0 PATIENT HAD A STROKE WITHIN THE LAST 3 MONTHS OR IS SUFFERING ONE

***PHYSICIAN ATTENDING THE PATIENT:***

***UNIT WHERE THE PATIENT IS HOSPITALISED: UNIT PHONES***

***ATTENDING PHYSICIAN'S EMAIL:***

## Annex 3: FUNCTIONAL ASSESSMENT AND QUALITY OF LIFE SCORE BEFORE STARTING NIV

WEIGHT: SIZE: BMI:

NECK CIRCUMFERENCE……(cm) ABDOMINAL CIRCUMFERENCE…………(cm)

FEV1 POST BETA 2 (L)……… FEV1 PERCENTAGE OF THEORETICAL: PERCENTAGE OF CHANGE WITH BETA2:

FVC POST-BETA2……………….FVC PERCENTAGE CHANGE: FEV1/FVC POST BETA2…………………

WALK TEST (METRES TRAVELLED):

PERCENTAGE OF METRES TRAVELLED RELATIVE TO EXPECTED:

MIP (cmH_2_O): MEP: 14.9.7 PERCENTAGE OF THE THEORETICAL:

CHEST X-RAY/CHEST CT SCAN: CANCER YES/NO

ABGs AT THE END OF TRAINING pH: PCO_2_: PO_2_: FIO_2_: HCO^-3^ ST: QUALITY OF LIFE SCORE (VALUE): APGAR (value):

BASELINE BODE SCORE (COPD):

**NUMBER OF HOSPITALISATIONS IN THE LAST 2 YEARS: NUMBER OF DAYS HOSPITALISED IN THE LAST 2 YEARS:**

***ANNUAL DISTRIBUTION BY AREA WHERE THE PATIENT WAS HOSPITALISED:***

**ICU:**  **DAYS/YEAR……………..**

**INTERMEDIATE: DAYS/YEAR**

**WARD ; DAYS/YEAR**

**MEDICINES FOR REGULAR USE (INDICATE AT LEAST TWO): BECLOMETHASONE, BUDESONIDE, FLUTICASONE, SPIRIVA, SALBUTAMOL, THEOPHYLLINE, PREDNISONE, ATROVENT**

| SUPPORT AND EQUIPMENT (INDICATE) BIPAP: | BIPAP+O_2_: | BIPAP WITH AVAPS: |
| --- | --- | --- |
| AVAPS+O_2_ |  |  |
| IPAP EPAP RR Ti | Ramp |  |

**BIPAP**

SUPPORT VIA: <A>(1 MASK, 2 TRACHEOSTOMY)

TYPE OF MASK OR ACCESSORY (1 NASAL, 2 ORONASAL, 3 FULL-FACE, 4 NASAL PRONGS, 5 ORAL DEVICES, 6 SNORKEL)

AVNIA RESPONSIBLE PHYSICIAN: (1 CESAR MAQUILON, 2 MONICA ANTOLINI) PHYSICAL THERAPIST EVALUATOR (OSVALDO 1, CRISTIAN 2)

NURSE EVALUATOR (PAOLA 1, KRISHANA 2, JUAN 3)

**Annex 4: INFORMED CONSENT FOR PATIENT AND/OR FAMILY MEMBER OR CARER**

"Home non-invasive Ventilation Programme in Adults"

AVNIA

Please read this document **carefully**. Information is herein provided necessary to decide whether you or next of kin should participate in the Home non-invasive Ventilation Programme.

General Background:

Non-Invasive Ventilation helps patients who have respiratory weakness due to various diseases such as muscular, neurological, spinal malformations, sleep apnoea, or chronic lung diseases during wakefulness or sleep.

Chronic hypoventilation is a condition progressively causing health deterioration.

The Government of Chile through the Ministry of Health has created a National Programme intended to provide the necessary equipment that together with supervision by health professionals, consisting of physicians, nurses, and physical therapists, will help you and your family to provide the non-invasive ventilation treatment needed.

BIPAP devices, used with a nasal or oronasal mask during the day or night, help maintain efficient breathing. This equipment significantly contributes to alleviating the symptoms related to your disease. BIPAP can be safely used by applying positive pressure during inspiration and expiration through a nasal or oronasal mask attached to your face.

The objective of this PROGRAMME is to use a BIPAP device to deliver Non-Invasive Ventilation (NIV) through a nasal or oronasal mask in patients with neuromuscular diseases, kyphoscoliosis, lung or respiratory diseases for a MINIMUM period OF 4 HRS PER DAY, relieve respiratory symptoms, provide rest of the respiratory muscles, decrease the sensation of shortness of breath, and finally decrease the frequency of hospitalisations, helping to improve the quality of life.

If you and your family agree to comply with the indications given by the health care team to be treated with non-invasive ventilation, our work team will be fully engaged to ensure you receive appropriate treatment for your illness under our supervision.

The organisation modality to grant the treatment and benefits you need consists of the delivery of the equipment (BIPAP) on bailment for home use with supervision by physical therapists and nurses in primary health care. This means the Health Service will deliver to you. temporarily, but for the entire period necessary, a BIPAP device and a set of complementary items (corrugated tubing, mask, attachment harnesses, and equipment filters) allowing to treat you or your family member with non-invasive ventilation.

On the other hand, you agree to take responsibility for the care of the equipment and to comply with the treatment indicated by the health team that will supervise it.

The selection criteria for admitting patients have been defined by a technical group and are based on the Technical Standards "Non-Invasive Ventilation Programme in Primary Health Care".

NIV treatment will be implemented by you or a family member and will be supervised by a health team specialised in the subject, which will evaluate the progression of your disease, provide training and monitor respiratory function through tests such as pulse oximetry, spirometry (forced inspiration and expiration through a mouthpiece) and study of muscle strength. Moreover, adherence to the use of the equipment will be monitored by downloading data from the memory card inserted into the equipment.

A clinical record will be filled out for each patient outlining the most relevant facts related to the patient's disease history. These data and your identity will remain confidential.

**What unwanted situations could happen? (Adverse events)**

Using BIPAPs for more than 12 hours or by masks held with undue force to the patient's face can cause skin lesions, on pressure areas such as the nasal bridge, as well as distension of the stomach, vomiting and infrequently lung rupture (pneumothorax). You will be trained to recognise these complications and other situations deviating from normal, which will be specified in a written plan for respiratory exacerbations or contingencies, non-respiratory events, and equipment failure.

What advantage or benefit does participation in this Programme represent for the patient?

You will receive the necessary equipment to be treated with NIV and will participate in personalised follow-up by the health team.

It should be noted you will be treated according to the usual technical and ethical standards of the service, requesting the usual tests according to your illness and consistent with your clinical progression.

**Confidentiality**:

Information about your medical history will be kept confidential to the extent permitted by law. This Programme ensures the rights and well-being of the participants are protected, warranting diagnostic, therapeutic, and follow-up actions are performed ethically.

The evaluation of the results of this Programme may be presented at medical meetings and in publications, and in no case will the identity of the patients who participated be revealed.

**Queries**:

Once you have decided to participate, you may contact the team of professionals of this Programme for questions or doubts related to the Programme or its procedures.

**Voluntary participation**:

The decision to participate in this programme is entirely voluntary. You can refuse it. This will not affect the benefits to which you may be entitled and the physician will advise you of the alternative treatments available.

If you decide to participate in the Programme, you will be given a copy of this information and asked to sign this Informed Consent.

If you decide not to participate, you will receive treatment as directed by your treating team.

**INFORMED CONSENT**

Title of the Programme:

"Non-Invasive Ventilation Programme in Primary Health Care".

It has been clearly explained to me and I have understood the essence and what my participation in this programme entails. I understand participation is voluntary and not participating will have no impact on the quality of medical care. I understand home treatment is my responsibility; however, I understand this Programme will provide me with the necessary equipment, supervision, training, and monitoring by the health care team. I have been given a copy of this form.

I……………………………………………………………………………………………

NAME SURNAMES SIGNATURE

give my authorisation to participate in this Programme.

.

Date: ……………

Address: ……………………………............................................................Phone: …………

I………………………………………………………………………...family member and/or responsible carer

NAME SURNAME SIGNATURE

My relationship with the patient is…………………………………………………………….I agree and under

my responsibility authorise the participation of my family member or person in my care in this Programme.

Date: …………………

Address: ……………………………............................................................Phone: …………

I confirm that I have explained all the necessary information about the Programme.

(to be completed by the nurse, physical therapist, or physician witnessing the consent)

Name: ………………………………………………………………

Signature: …………………………………………………………......

Position: ……………………………………… Date: ...............................

**Annex 4: EQUIPMENT LOAN (BAILMENT) CONTRACT**

In the City of , the of of 20 , between the Health Service

, legal entity governed by public law, domiciled in , represented by its Director MD. , Chilean, physician , National Identity Card Nº , both with the same address, hereinafter "The Service" by one party; and by the other Mr(Ms) , national identity card N° (nationality) , (marital status) , (profession/activity) , with residence in this city,

, commune of , hereinafter the "Baylor", the following has been agreed:

**First**: By medical prescription of MD. (a) the Patient

, Resident at ,

Telephone

ID

Participates in the National Programme of Home Non-Invasive Ventilation in Adults (AVNIA) implemented by the Respiratory Health Unit of the Primary Care Division of the Undersecretary of Care Networks of the Ministry of Health, hereinafter “The Programme”.

**First**: By medical prescription of MD. (a) the Patient

, Resident at … ,

Telephone ID. participates in the Home Non-Invasive Ventilation National Programme for Adults (AVNIA) implemented by the Respiratory Health Unit of the Primary Care Division of the Ministry of Health, hereinafter “The Programme”.

**Second**: To develop the Programme in the patients' homes, the Service will work with Primary Health Care Teams that will be responsible for the installation, start-up of the equipment, education and training of the Bailee, as well as overseeing the actions specified in the Programme.

**Third**: Within the framework of the Programme, the Bailee has received from the Service, as a bailment, the bi-level pressure ventilator and its set of complementary items (hereinafter the "EQUIPMENT") described next:

| Equipment Description | Serial Nª | Inputs of each equipment | Others |
| --- | --- | --- | --- |
|  |  | (Set of complementary items) |  |
|  |  |  |  |
|  |  |  |  |
|  |  |  |  |
|  |  |  |  |
|  |  |  |  |

**Fourth**: By this act, the Service delivers as a bailment to the Bailee, who accepts it, the EQUIPMENT described in the preceding clause, under the terms and conditions set forth in this contract. The aforementioned bailment is agreed under the terms of article 2.194 of the Civil Code, i.e., the Service reserves the right to request the return of the EQUIPMENT to the Bailee at any time. Notwithstanding the foregoing, it is stated this contract will terminate and the Bailee shall return the EQUIPMENT in the event the Patient is withdrawn or stops participating in the Programme.

**Fifth**: The Bailee declares having received the EQUIPMENT described above in good condition and to its entire satisfaction at its residence, on the date of

of 20..., It further declares that after being properly installed by Service personnel, the EQUIPMENT was fully operational and that its operation was tested in its presence by Service personnel.

**Sixth**: At the time of EQUIPMENT delivery, the Bailee and the Patient were trained by Service personnel in EQUIPMENT handling and, in the care, and precautions required to handle the EQUIPMENT and its accessories.

**Seventh**: The Bailee declares it was informed by the Service personnel about the risks the incorrect or careless handling of the EQUIPMENT and its accessories could cause. Likewise, it declares having received written material (brochures, user manual, etc.) with information about the EQUIPMENT and its correct handling.

**Eighth**: The Bailee declares and acknowledges the EQUIPMENT received on bailment, is a piece of therapeutic equipment used in people, so to avoid damage to the Patient and the EQUIPMENT it must be handled in strict accordance with the rules and procedures communicated by Service personnel and are included in the written material delivered to you. In particular, the Bailee acknowledges it must comply with the following precautions:

1.- The Programme has been designed for the use of the equipment during the patient's sleep or wakefulness and for a period indicated by the attending physician, necessary to minimise the risks derived from the prolonged use of pressure systems on the support areas of the mask.

2.- The equipment requires for its use on the patient, a nasal or oronasal mask fixed to the patient using an attachment harness and connected to the equipment by corrugated tubing. The complementary set includes these three accessories.

ASSOCIATED RISKS

1. A tight fit of the mask to the patient's face can cause skin lesions on pressure areas, mainly the nasal bridge.
2. Flow leakage from the mask into the eyes can cause conjunctival irritation.
3. Excessive air leakage from the mask or any discontinuity of corrugated tubing will generate greater flow through the equipment to maintain pressure; this can be associated with distention of the stomach and vomiting, intolerance of the patient to the treatment and infrequently lung rupture (Pneumothorax).
4. The use of BIPAPs for more than 10 hours can cause skin lesions on pressure areas, usually the nasal bridge, distention of the stomach and vomiting and infrequently lung rupture (Pneumothorax).
5. You will be trained to recognise these complications and other situations deviating from normal; these will be specified in a written plan for respiratory exacerbations or contingencies, non-respiratory events, and equipment failure

Flow Generators (BIPAP) - RECOMMENDATIONS:

1. Handle the EQUIPMENT with care since it is electrical equipment.
2. Keep the EQUIPMENT connected to a properly installed electrical outlet. DO NOT connect to improperly installed or damaged outlets. DO NOT use extension cords (except extension cords supplied with EQUIPMENT) or electrical adapters. DO NOT use EQUIPMENT if the plug or cord is damaged.
3. While the EQUIPMENT is working, it must ALWAYS be kept in a HORIZONTAL position (lying down), NEVER place it in a vertical position because it would deteriorate its operation.
4. AVOID bumping and dropping of EQUIPMENT.
5. Keep the reusable filter clean. Filter cleaning must be performed according to the periodicity and instructions contained in the brochure and informed by Service personnel. It is important to unplug the EQUIPMENT before cleaning. NEVER install a wet filter.
6. DO NOT operate the EQUIPMENT without fitting the filter.
7. Do not smoke in the vicinity of the EQUIPMENT.
8. EQUIPMENT must NOT be used as a table. DO NOT place objects, glasses or jugs with liquids on the EQUIPMENT as spilling liquids on the EQUIPMENT may damage it.
9. Check the air intake or inlet, where the external filter is located, is always CLEAR BY 50 CM. OF THE WALL and is not obstructed by curtains or blankets, as it may hinder or impair EQUIPMENT performance.
10. Do not install the EQUIPMENT in small or enclosed spaces (e.g., closet), but rather in places where there is air circulation, although it is not necessary to keep the windows open in winter.
11. Keep the EQUIPMENT away from stoves, cookers, ovens, electrical appliances, especially toasters, hairdryers, heaters, in general from any source of combustion.
12. DO NOT USE or store the EQUIPMENT near combustible materials such as oils, fats, aerosols (spray), lotions, solvents, paraffin, alcohol, among others.
13. DO NOT handle the EQUIPMENT with your hands with creams, fats, oils or fuels of any kind (e.g., paraffin, alcohol, etc).
14. DO NOT store or operate the EQUIPMENT in WET places such as bathrooms, balconies, etc.
15. DO NOT allow the EQUIPMENT to receive direct sunlight, heat damages the EQUIPMENT.
16. DO NOT allow children or untrained personnel to operate the EQUIPMENT.

If you have any problems or questions regarding the equipment, call the PHC Health Team assigned to you.

Interfaces (Masks), Attachment Harness, and Corrugated Tubing- RECOMMENDATIONS

- 1. The items listed should be handled by the carer and patient with clean, dry hands.
  2. The mask and corrugated tubing must be cleaned according to the periodicity and instructions contained in the brochure and informed by the Service personnel.
  3. It is important doing this away from the EQUIPMENT so as not to expose it to moisture or direct contact with water.
  4. Cleaning should be done only with soap and water.
  5. These items should not be exposed to fire, heat sources or direct and prolonged sunlight. Nor should they be boiled or heated. Corrugated tubing should not be cut.
  6. When the patient is using these items, they should not be handled with hands with creams, oils or fuels of any kind (e.g., grease, oil, paraffin, etc.), due to the risk of combustion.

**Ninth**: The Service assumes no responsibility for events arising from improper handling of the EQUIPMENT or use in contravention of the PRECAUTIONS and safety measures outlined in this Contract and in the EQUIPMENT safety manuals and brochures provided to the Baylee.

**Tenth**: If the Service personnel detects that in handling the EQUIPMENT the Bailee has not complied with the PRECAUTIONS and safety measures described in clause Seven above, this situation will be reported to the Respiratory Health Unit of the Division of Primary Care of the Undersecretary of Care Networks of the Ministry of Health so that it may proceed to remove the Patient from the Programme.

**Eleventh**: The Bailee may not lease, encumber or constitute any right over the EQUIPMENT and its accessories, nor assign its use, enjoyment or mere possession under any title.

**Twelfth**: The EQUIPMENT may only be used by the Patient specified in clause One of this contract and according to the treatment prescribed by the treating physician. It is hereby stated that the non-invasive ventilation treatment prescribed for the Patient is the sole responsibility of the treating physician, and the Service, through the Respiratory Health Unit of the Primary Care Division of the Undersecretary of Care Networks of the Ministry of Health, limits itself to install and regulate the EQUIPMENT according to the conditions established by said professional.

**Thirteenth**: The EQUIPMENT will be maintained by the Bailee only and exclusively at the address indicated in the appearance.

**Fourteenth**: The Bailee undertakes to take care of the EQUIPMENT in such a way as to prevent damage to the EQUIPMENT or any of its parts and accessories.

**Fifteenth**: The Baylee shall inform the Service through the Programme Coordinator of the Respiratory Health Unit of the Division of Primary Care of the Undersecretary of Care Networks of the Ministry of Health promptly and on time of any problem arising about the ownership, possession, and use of the EQUIPMENT, especially including claims from third parties claiming rights of any kind over the EQUIPMENT and shall indicate to third parties intending to attach liens, precautionary measures or similar that the EQUIPMENT belongs solely and exclusively to the Service. The Bailee will indemnify the Service for any damages suffered as a result of the breach of this obligation.

**Sixteenth**: The Bailee undertakes to restore the EQUIPMENT covered by this contract as soon as it is requested by the Service in the same condition in which it was received.

**Seventeenth**: For all purposes arising from this contract, the parties submit to to the jurisdiction of the courts of the city and commune of .

**Eighteenth**: This agreement is signed in three copies of the same tenor and date, two being in the possession of the Service and one in the possession of the Bailee.

Mr (Mrs).................................................

Tax ID#

Mr (Mrs).................................................

Tax ID#

Health Service Director.......................

##

## TECHNICAL GROUP-STANDARDS

Sandra Navarro Respiratory Health Unit MINSAL

Mónica Antolini Toledo Technical Reference

Cesar Maquilón Ortiz Technical Reference

**Pulmonologists' Advisory Committee for Adults**

Ricardo Sepúlveda National Thorax Institute

Carlos Peña Hospital Complex San Borja Arriarán

Luis Soto National Thorax Institute Francisco Arancibia National Thorax Institute

Felipe Aller National Thorax Institute

Fernando Descalzi Hospital Padre Hurtado, and German Clinic [*Clínica Alemana*]

Jorge Villalobos Hospital Sótero del Río

Orlando Díaz Pontifical Catholic University of Chile [*Pontificia Universidad Católica De Chile*]

Guillermo Montiel Respiratory Rehabilitation Hospital María Ferrer [*Hospital de Rehabilitación Respiratoria María Ferrer*].Buenos Aires Argentina

**Neurologists' Advisory Committee**

Sergio Castillo Hospital del Salvador

**Physical therapists' Advisory Committee**

Gerardo Ferrero Respiratory Rehabilitation Hospital María Ferrer [*Hospital de Rehabilitación Respiratoria María Ferrer*]

Osvaldo Cabrera National Thorax Institute

Cristian Olave National Thorax Institute

**References**

1. Maquilón C, Non-Invasive Mechanical Ventilation in decompensated COPD patients [Ventilación Mecánica no Invasiva] en pacientes con EPOC descompensada]. Rev Chil Enf Respir 2002; 18:169-174
2. Díaz O, Begín P, Torrealba B,Jover E, Lisboa C. Effects of noninvasive ventilation on lung hyperinflation in stable hypercapnic COPD. Eur Respir J 2002; 20: 1490-1498.
3. Clini E, Sturani C, Rossi A, et al. The Italian multicentre study on noninvasive ventilation in chronic obstructive pulmonary disease patients. Eur Respir J 2002; 20: 529-538.
4. Prado F, Boza ML, Koppmann A. Paediatric nocturnal domiciliary non-invasive ventilation [Asistencia ventilatoria no invasiva domiciliaria nocturna en pediatría]. Rev Chil Enf respir 2003; 19:146 - 154.
5. Sánchez I, Valenzuela A, Bertrand P, Álvarez C, Holmgren N, Vilches S, Jerez C, Ronco R. Domiciliary ventilation in children with chronic respiratory failure. Clinical experience. [Apoyo ventilatorio domiciliario en niños con insuficiencia respiratoria crónica. Experiencia clínica]. Rev. Chil. Pediatr 2002; 73:51-55.
6. Bertrand P, Felhmann E, Lizama M, Holmgren N, Silva M, Sánchez I. Home ventilatory support in Chilean children: 12 years of experience [Asistencia ventilatoria domiciliaria en niños chilenos: 12 años de experiencia]. Arch Bronchoneumol 2006;46(4):165-70.
7. Wijkstra P, Lacasse Y, Guyatt G, Casanova C, Gay P, Meecham Jones J, Goldstein R. A Meta-analysis of nocturnal Noninvasive Positive Pressure Ventilation in Patients with Stable COPD. Chest 2003;124.337-343
8. ANTADIR. Therapie, 2001 Mar-Apr; 56(2):143-9
9. Sugerman H, Fairman P, Baron P, Kwentus J. Gastric Surgery for Respiratory Insufficiency of obesity. Chest 1986; 90:81-86
10. Kessler R, Chaouat A, Schinkewitch P, Faller M, Casel S, Krieger J, Weitzenblum E. The obesity-Hypoventilation Syndrome Revisited. Chest 2001; 120:369-376.
11. Chatwin M, Nickol A, Morrel M, Polkey M, and Simonds A. Randomised trial of inpatient versus outpatient initiation of home mechanical ventilation in patient with nocturnal hypoventilation”. Respiratory Medicine (2008) 102; 1528-1535.
12. Chilean consensus on non-invasive ventilation [Consenso Chileno ventilacion no invasiva]. Revista Chilena de enfermedades respiratorias.Vol 24 N° 3, 2008.
